# Architecture, Chromatin and Gene Organization of *Toxoplasma gondii* Subtelomeres

**DOI:** 10.3390/epigenomes6030029

**Published:** 2022-09-15

**Authors:** Susana M. Contreras, Romina T. Zambrano Siri, Elías M. Rivera, Constanza Cristaldi, Laura Kamenetzky, Kami Kim, Marina Clemente, Josefina Ocampo, Laura Vanagas, Sergio O. Angel

**Affiliations:** 1 Laboratorio de Parasitología Molecular, Instituto Tecnológico Chascomús (INTECH), Universidad Nacional de General San Martín (UNSAM), Consejo Nacional de Investigaciones Científicas y Técnicas (CONICET), Chascomús 7130, Argentina; 2 Instituto de Investigaciones en Ingeniería Genética y Biología Molecular “Dr. Héctor N. Torres” (INGEBI-CONICET), Buenos Aires C1428ADN, Argentina; 3 Laboratorio de Genómica y Bioinformática de Patógenos, iB3|Instituto de Biociencias, Biotecnología y Biología traslacional, Departamento de Fisiología y Biología Molecular y Celular, Facultad de Ciencias Exactas y Naturales, Universidad de Buenos Aires, Buenos Aires C1428EHA, Argentina; 4 Department of Internal Medicine, Morsani College of Medicine, University of South Florida, Tampa, FL 33602, USA; 5 Laboratorio de Molecular Farming y Vacunas, Instituto Tecnológico Chascomús (INTECH), Universidad Nacional de General San Martín (UNSAM), Consejo Nacional de Investigaciones Científicas y Técnicas (CONICET), Chascomús 7130, Argentina

**Keywords:** *Toxoplasma*, telomere, subtelomere, chromatin, multigene family, environmental adaptation

## Abstract

Subtelomeres (ST) are chromosome regions that separate telomeres from euchromatin and play relevant roles in various biological processes of the cell. While their functions are conserved, ST structure and genetic compositions are unique to each species. This study aims to identify and characterize the subtelomeric regions of the 13 *Toxoplasma gondii* chromosomes of the Me49 strain. Here, STs were defined at chromosome ends based on poor gene density. The length of STs ranges from 8.1 to 232.4 kbp, with a gene density of 0.049 genes/kbp, lower than the Me49 genome (0.15 kbp). Chromatin organization showed that H3K9me3, H2A.X, and H3.3 are highly enriched near telomeres and the 5′ end of silenced genes, decaying in intensity towards euchromatin. H3K4me3 and H2A.Z/H2B.Z are shown to be enriched in the 5′ end of the ST genes. Satellite DNA was detected in almost all STs, mainly the sat350 family and a novel satellite named sat240. Beyond the STs, only short dispersed fragments of sat240 and sat350 were found. Within STs, there were 12 functional annotated genes, 59 with unknown functions (Hypothetical proteins), 15 from multigene FamB, and 13 from multigene family FamC. Some genes presented low interstrain synteny associated with the presence of satellite DNA. Orthologues of FamB and FamC were also detected in *Neospora caninum* and *Hammondia hammondi*. A re-analysis of previous transcriptomic data indicated that ST gene expression is strongly linked to the adaptation to different situations such as extracellular passage (evolve and resequencing study) and changes in metabolism (lack of acetyl-CoA cofactor). In conclusion, the ST region of the *T. gondii* chromosomes was defined, the STs genes were determined, and it was possible to associate them with high interstrain plasticity and a role in the adaptability of *T. gondii* to environmental changes.

## 1. Introduction

Heterochromatin, densely packed DNA with low transcriptional activity, constitutes the major component of most eukaryotic nuclei and is essential for maintaining genomic stability. In most eukaryotes, heterochromatin is concentrated in the pericentromeric, telomeric, and subtelomeric regions and is highly enriched in repetitive sequences [1]. Subtelomeres (STs) are chromosome regions adjacent to telomeres and are rich in satellite DNA, pseudogenes, and, sometimes, complete genes that are generally species-specific [2,3,4,5]. In addition, the heterochromatin that forms the ST is characterized by low nucleosome occupancy and the presence of repressive histone marks such as H4K20me3, H3K9me2/3, and H3K27me3. Those marks, in turn, recruit heterochromatin factors such as HP1 and deacetylases such as Sir2, which target H3 and H4 [6,7,8,9].

One of STs’ functions is to contact the chromosomes with the nuclear envelope [10]. Another very important function is to protect euchromatin from the invasion of telomeric heterochromatin to safeguard gene expression [11]. STs are also involved in telomere maintenance, telomere length regulation, and late DNA replication [12,13,14].

STs are difficult to define because these gene-poor regions evolve quickly. The extraordinary instability of STs allows rapid adaptation to new niches, resulting from the high rate of gene duplication, followed by the functional divergence of the alleles [15]. Subtelomeric genes often comprise groups of genes associated with a determined metabolic pathway, virulence, and pathogenicity [15,16,17,18,19,20,21,22]. The genes of the subtelomeric regions, including those whose expression is associated with certain environmental conditions, are under the telomeric position effect (TPE), which keeps chromatin silenced [4,12,23]. The role of chromatin is essential for the functions of the STs. For example, the subtelomeric regions of *Plasmodium* and the regulation of its antigenic variation genes are subject to correct chromatin modulation by different factors, including histone marks and histone variants H2A.Z and H2B.Z [24].

*Toxoplasma gondii* is an apicomplexan protozoan parasite with an infection rate close to one-third of the human population [25]. It is capable of infecting all warm-blooded animals and all nucleated cells. It presents a complex life cycle with several stages, which guarantees its success in dispersion. All this implies high flexibility to develop in the different environments in which it is dispersed. Despite the role that STs could have in this adaptation to different environments, very little is known about them. ST regions were detected and characterized in nine out of the 14 *Toxoplasma* chromosomes, now revised to 13 [26,27], based on chromosome end similarities and designated telomere-associated sequences (TAS) [28]. *T. gondii* Chromo1 protein (a heterochromatin protein 1 or HP1-like protein) was found to be associated with the peri-centromeric regions and the repeats present at the end of chromosomes Ia and IX in the nuclear periphery [29]. In a separate set of ChIP-qPCR experiments, an enrichment of the heterochromatin marks H4K20me1 and H3K9me1 with the subtelomeric repetitive element sat350 was also detected [30]. Genome-wide analysis showed that H2A.X and H3.3 are also enriched at the chromosome ends [31].

A *T. gondii* multigene family (FamC) was detected at *T. gondii* STs [28]. FamC genes were named *Toxoplasma* specific factors (TSF) without an attributable function [32]. Recently, Lorenzi et al. [33] classified this family and other multigene families of *Toxoplasma* within the set of proteins important for the pathogenesis and evasion of the immune system response (FamA, FamB, FamD, FamE, Rop, Mic, Gra, SRS and SAG). FamA, FamB, FamC, FamD, and FamE are predicted to be integral membrane proteins but differ in motif organization and length [32,33]. These genes were detected in the genomes of *T. gondii*, *Hammondia hammondi* and *Neospora caninum*, except FamC, which was detected only in *T. gondii* and *H. hammondi*. The Fam genes were not detected in other Apicomplexan protozoa [33].

A more in-depth sequence of the *T. gondii* genome is now available, allowing the subtelomeric regions to be re-analyzed in detail. Here, we defined STs of 13 *T. gondii* chromosomes based on gene density. The defined STs were analyzed in terms of their chromatin architecture and the presence of satellite DNA. We also identified and characterized subtelomeric genes based on gene description, synteny, chromatin-associated status, and transcriptional and proteomic evidence. Finally, we analyzed the expression profile of ST genes in different situations: extracellular passage transcriptome from an evolve and resequencing study, an approach to investigate the genomic responses to selection during experimental evolution [34], and changes in metabolism based on ΔACL/iΔACS transcriptome data [35].

## 2. Results

### 2.1. Defining T. gondii STs

**Figure 1:**
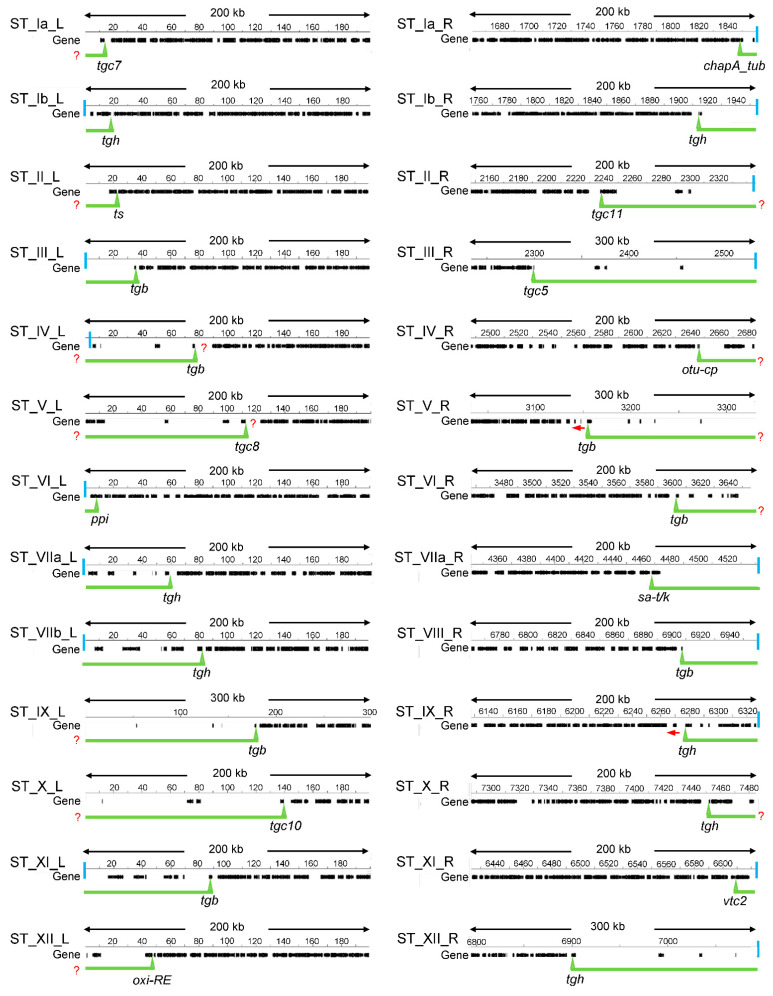
Definition of subtelomeric regions based on gene density. Regions with low gene density were defined manually by scanning the chromosome ends. The border (triangle) was defined from the first gene flanking the euchromatin-compatible region. The vertical light blue line indicates the presence of telomeric repeat. The red ? indicates the possibility that the ST region extends beyond the defined one. The red arrow indicates the orientation of the gene. Numbers above the line indicate the position in chromosomes (in kb). Me49 data was obtained from ToxoDB versions 53 and 57. No difference was observed. Version 53 of the reference strain ME49 was used.

The identification of *T. gondii* STs based on the search for conserved regions only detected nine STs out of a possible 26 [28], indicating that this strategy was insufficient. In yeast, STs were characterized on the basis of their poor gene density, compared to the high gene density-associated euchromatin [15]. Similarly, we defined the STs of the 13 *T. gondii* chromosomes by scanning the chromosome ends to detect gene density regions. In euchromatin, genes are separated among them by a short intergenic region. In *T. gondii*, the general intergenic region was observed to be of ~2000 bp, similar to what was proposed by the genome project (3404-bp) and also in *N. caninum* (3091 bp) [26,36]. Taking into account that the ST genes would be unrepresentative, we assume that these values are close to what is expected as a mean for intergenic distance in *T. gondii* euchromatin. For this reason, here we used an intergenic distance of 3500 bp between consecutive genes initiating a long genic array region. Near 200–300 kbp were scanned to detect gene poor regions from euchromatin (Figure 1). The boundaries of the STs were defined by the 5′ end of the first gene that is at the border of a region compatible with euchromatin as defined above. In cases where two contiguous genes were starting euchromatin, we chose the 5′ end of the one oriented towards the telomere. In cases where an ST FamC gene was immediate to the euchromatin, we took this as the border, as had been proposed [28]. Similarly, we selected the ST FamB gene as a border when it was immediate to euchromatin based on studies performed here (see below). With few exceptions, ST regions were easily defined with manual scanning. In the case of ST_Ib_L, TGME49_207370 was selected because the two upstream hypothetical genes have no evidence of expression. In the cases of ST_V_R and ST_IX_R, the upstream genes of selected border genes are oriented towards the centromere. It is important to notice that ST border genes only delimit the euchromatin from the gene-poor region, although it is unclear if they are subject to TPE. In this work, we have considered all of these genes associated with STs. On the other hand, it could be possible that in some cases STs extend beyond the border gene, as it is shown for ST_IV_L and ST_V_L (Figure 1).

The proposed STs and their most relevant characteristics are summarized in Table 1. The putative STs for all the *T. gondii* chromosomes could be established, which have a length ranging from 8.1 to 232.4 kbp (average 76.3), with a gene content ranging from one to eight per ST (average 3.7), and a GC content averaging 52.37%, similar to GC genomic content (Table 1). The ST gene density was calculated as the number of genes/kbp, resulting in 0.049, a low value compared to that observed for the general Me49 chromosomes gene density (0.16 genes/kbp).

Only 16/23 STs were associated with a *T. gondii* telomeric TTTAGGG repeat, suggesting that in such cases, the ST could be longer toward the telomere end (Figure 1). In 12 of the STs, we defined the ST border as a gene from FamB or FamC families, whereas 14 STs were delimited by single copy genes, either hypothetical or functional annotated genes (Table S1).

### 2.2. ST Chromatin Architecture

**Figure 2:**
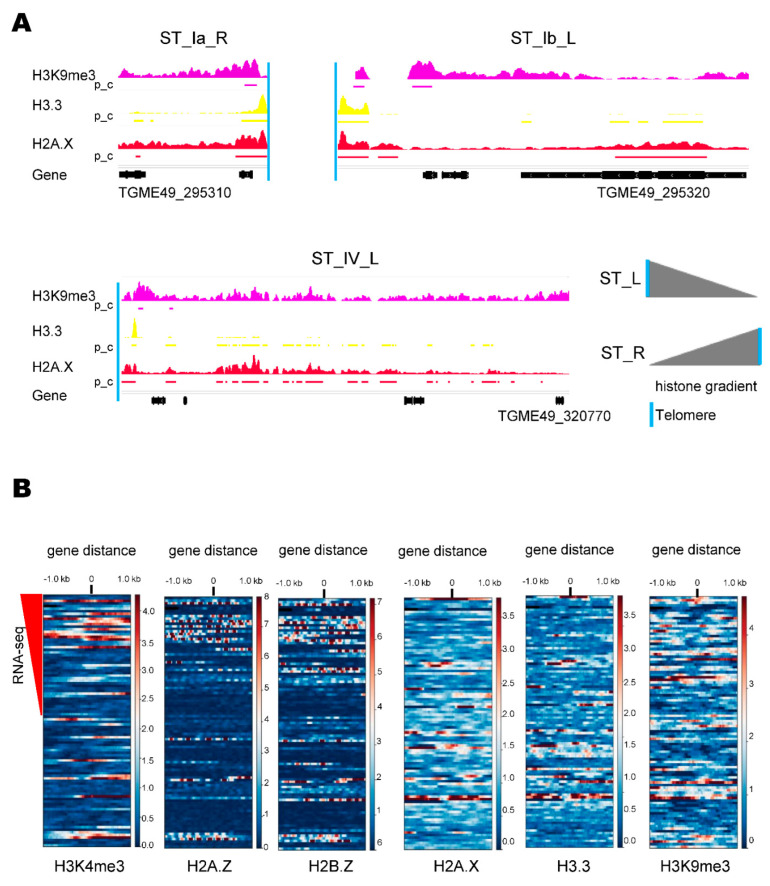
*Toxoplasma gondii* ST chromatin architecture. (**A**) Examples of H3K9me3 (violet), H3.3 (yellow), and H2A.X (red) distribution in *T. gondii* STs. The occupancies of histone marks were explored with the Integrated Genomics Viewer (IGV). Scale: H3K9me3, 0–4.21; H3.3, 0–1.76 and H2A.X, 0–2.05. Below each histone coverage track, the positions of the histone peaks identified by MACS2 peak_calling (pc) analysis are shown. The lower black bars correspond to the presence of genes along the chromosomes. The vertical light blue line indicates the presence of telomeric repeat. Below: the geneID of the STs border some genes. Version 53 of the reference strain ME49 was used. Gray triangles indicate the observed gradient of histone marks along the ST from the telomere. (**B**) Heatmaps showing occupancies of histone marks for the 97 ST genes sorted according to their levels of expression (from top to bottom). The reference point indicated by 0 represents the position of the 5′ UTR or the initiation codon ATG of the CDS. The color level indicates the occupancy of the histone, brown representing high intensity and blue representing minimum intensity. The red triangle indicates the descendent expression gradient measured by RNA-seq for 47/97 ST genes (see Table S3).

Histone marks and variants were analyzed in every chromosome. The global histone analysis showed clear peaks for H3K9me3, H3.3, and H2A.X at regions delimiting the ends of the chromosomes [31]. A detailed analysis of ChIP-seq assays (Table S2) showed that the intensities and/or peaks of these histones decreased from the chromosome end toward the centromere in most cases, being more evident for H3.3 and H2A.X (Figure 2A and Figure S1).

The histone H3K4me3 is a mark of transcription start sites (TSS)/promoters of active genes and colocalizes with histone variants H2A.Z and H2B.Z at almost all TSS/promoters of active genes [31,37]. Interestingly, it was suggested that the sole presence of H2A.Z/H2B.Z in a TSS is associated with the bivalent stage of chromatin. ST genes are subject to rapid expression in response to environmental changes, a condition that can be associated with bivalent promoters. Based on that, the presence of H3K4me3, H2A.Z, H2B.Z, H3.3, H2A.X, and H3K9me3 at the 5′ ends of the subtelomeric genes associated with their transcriptional activity was studied (Figure 2B). Only 39/97 ST genes showed RNA-seq evidence (Table S3). In the heat map analysis, the represented genes keep on the y-axis the same sorting as shown in the Table S3. In general, H3K4me3 colocalizes with H2A.Z/H2B.Z dimer at 5′ ends of active genes. There are seven cases of H3K4me3/H2A.Z and/or H2B.Z histones’ co-localization in genes without RNA-seq evidence, which may be associated with differences in the transcriptomic analysis. In addition, there were only two cases in which the dimer H2A.Z/H2B.Z was present at the 5′ ends of moderately active genes, suggesting that ST are not enriched in H2A.Z/H2B.Z-bivalent genes. H3.3 colocalizes with H2A.X, and both partially with H3K9me3, mainly at genes with intermediate or undetected expression.

### 2.3. T. gondii STs Are Enriched in Tandem DNA Repeat Elements

Long tandem repetitive elements (from 10 to hundreds of bp) that may be present in regions of several kbp, called satellite DNA, are located in heterochromatic regions such as STs [38]. To analyze the presence and composition of satellite DNA at *T. gondii* STs, a dot plot study was carried out. In this way, it was possible to detect regions of repetitive DNA in tandem, compatible with satellite DNA, in 18/26 STs (Table 1, Figure S2). Almost all of the repetitive elements do not have high identities (data not shown). However, the presence of sat350 and sat240, as major satellite DNAs of *T. gondii* STs, could be detected. Noteworthy, sat529, a centromeric 529-bp satellite DNA [27,39], was also detected in ST_IV_R.

**Figure 3:**
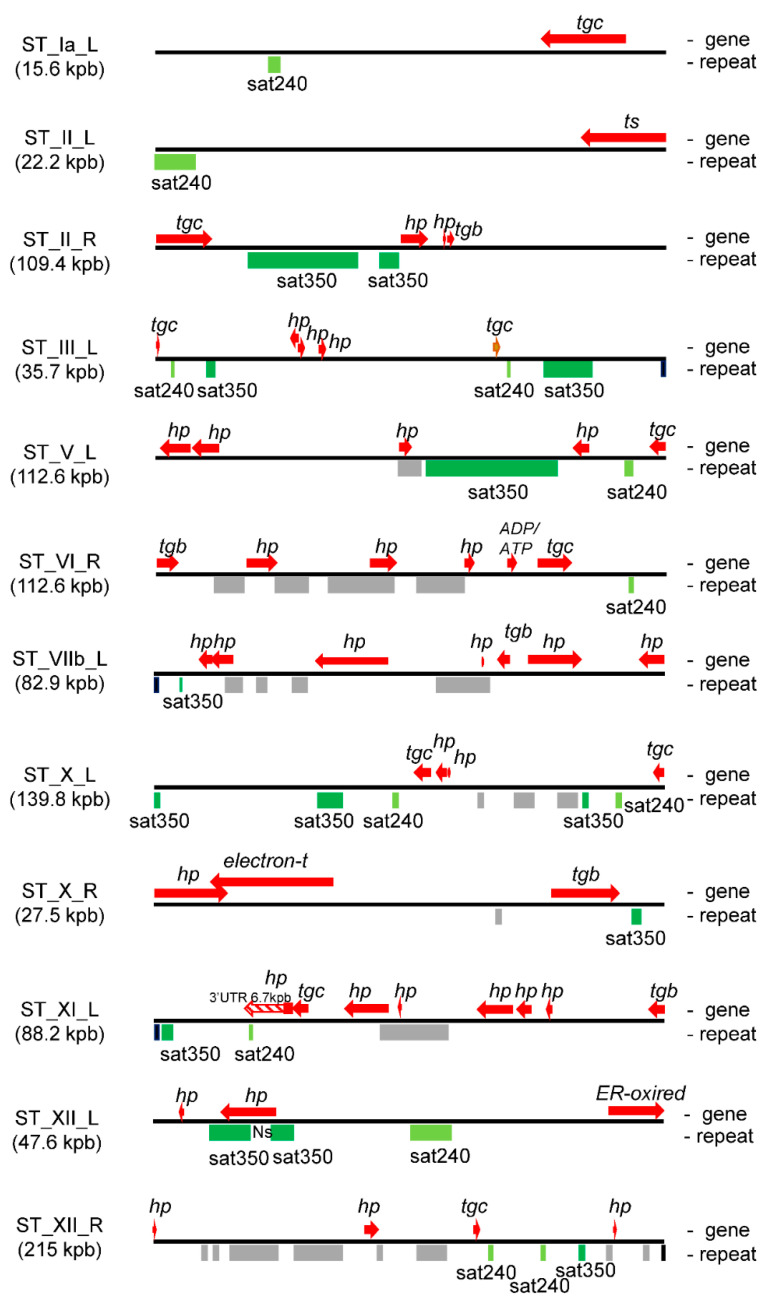
Arrays of genes and satellite DNA sequences present in STs. Based on the dot plot analysis and data from Table S1, a map of the position of each gene (top) and satellite DNA (bottom) was constructed for each ST. The figure shows a selection of STs that contain at least one sat240 and/or sat350. The length of satellite DNAs and genes are scaled for each ST. Note that each ST has a different scale according to its length. Sat350: green; sat240: light green; other satellite DNAs: grey. The names of the genes respond to the criteria listed in Table S1: Ns, track of N (any nucleotide) in the ST sequence, and genomic genes.

In order to determine whether sat350 and sat240 are also dispersed in non-subtelomeric regions of the *T. gondii* genome, identity screening was performed along all chromosomes using a dot plot analysis. In Figure S3, it is possible to observe that satellite DNA blocks containing sequences from the sat240 or sat350 families only appear in the STs of expected chromosomes and only in one case close to the centromeric region of chromosome IV (Figure S3). It is also possible to observe numerous small fragments (<100 bp) of both satellite DNAs, but mainly sat240, in internal regions of the chromosomes, compatible with euchromatin. Only a few 240 bp fragments for sat240 appear in euchromatin. This analysis indicates that sat240 and sat350 DNA satellite structures only appear in STs. Data were confirmed by Blastn searching at ToxoDB. The genome database only retrieved short sequences from both DNA satellites (data not shown). Analyzing the transcript database, only Sat240 retrieved two long sequences with significant similarity. One corresponded to the 3′UTR region of the ST gene transcript TGME49_307270, which has a long 3′ UTR region (Figure 3), and the other, with less similarity, to the 5′ UTR region of the TGME49_300980 gene (chromosome V), at 3012 bp from the ATG (data not shown).

### 2.4. Satellite DNA and Subtelomeric Genes

*T. gondii* STs present numerous predicted genes (Table S1), in addition to satellite DNAs. In order to know the distribution of these DNA elements, a map was generated for each ST with the sequences of the genes as they were annotated in ToxoDB and the sat350 sequences obtained in Figure S2. The vast majority of genes are positioned between satellite DNA blocks (Figure 3 and Figure S4). In fact, only six genes overlap with repetitive DNA, and only in one case with the sat350 element. It should be noted that the gene (TGME49_300790) that shares sequences with sat350 has a long *Ns* track in its sequence, which, in fact, separates the two sat350 blocks (Figure 3). Notably, 10 *tgc* genes (FamC family) were closely associated with sat240 and sat350 (Figure 3).

STs are very dynamic and unstable regions, and part of this dynamism is generated by DNA repeats. Consistent with this, Xia et al. [27] observed high plasticity in the copy number of sat350 among *T. gondii* strains and F1 progeny. To know if the dynamics can be extended to the genes present in the STs, an interstrain synteny analysis was carried out based on the presence of the genes enlisted in Table S1. The genome organization of 14 strains of *T. gondii* was retrieved from ToxoDB. Low synteny was considered when the analyzed ST gene was present in the same genome location only in six of the 14 *T. gondii* strains, or less, according to ToxoDB (Orthology and synteny section). Gene duplications and the presence of new genes were also included in some strains. The loss or gain of genes among strains was more evident in internal ST genes, mostly associated with the presence of satellite DNA, especially with sat240/sat350 (Table S1).

### 2.5. Features of ST Genes

**Figure 4:**
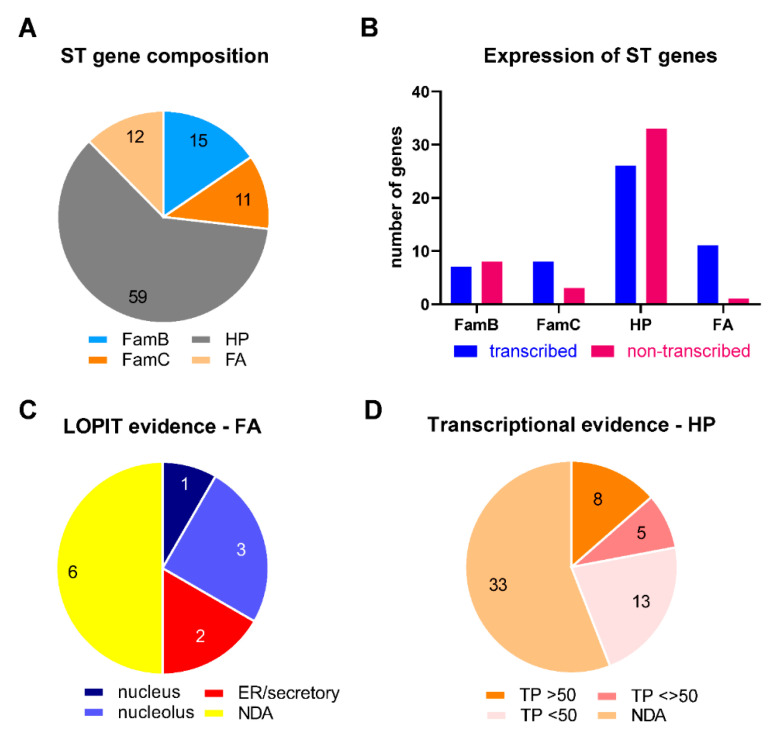
Analysis of genes detected in *T. gondii* STs (ST). The graphs were obtained from the data displayed in Table 2, Tables S1 and S4. HP: hypothetical protein. FA: functional annotated gene. (**A**) Gene composition of ST. The numbers represent the number of genes. (**B**) The number of genes showing transcriptional evidence in the ToxoDB database is based on the expression profiling of three archetypal lineage sources. The transcription expressed as percentages of gene expression were obtained for Me49 and RH strains. Other transcriptional evidence was not taken into account. (**C**) Subcellular localization of FA group genes according to a LOPIT analysis retrieved from ToxoDB. The numbers represent the number of genes. (**D**) Genetic evidence of hypothetical gene expression according to the level of transcription measured in each percentile (TP). Percentile values greater than 50 and less than 50 were retrieved from ToxoDB for Me49 and RH strains. When the levels were greater than 50 in one strain and less than 50 in another strain, they are shown as <>.

Ninety-seven genes were identified in the STs, which were referred to as functional annotated (FA), hypothetical (HP), multigene family FamB, and multigene family FamC. The main features of these genes are described in Table 2 and Table S4. Only 12 HP and two FA genes present paralogs, some of them also located at ST (Table S1). The vast majority of them encode HP proteins (Figure 4A). The *T. gondii* Me49 strain, of low virulence but with cystogenic potential both in vitro and in vivo, has been taken as a reference in ToxoDB. However, there are transcriptomic data for other strains of *T. gondii*. We decided to incorporate data from Me49 and the highly virulent, non-cystogenic RH strain in this analysis. Analyzing the ToxoDB database, we were able to detect evidence of transcription in 53.7% of subtelomeric genes at ToxoDB (Table 2 and Table S4) and 40.2% by RNA-seq database (Table S3). The analysis by gene group shows variation in the expression rate among them, being lower in the group of hypothetical genes and higher in the group of simple functional annotated (FA) genes (Figure 4B).

Twelve FA genes were identified in the STs; almost all showed evidence of transcription, in many cases with proteomic data (Figure 4B, Table 2). Most functional annotated genes presented expression levels below the 50th percentile, without differences between strains (Table 2). Eight had a negative phenotype score, suggesting that they could be essential for the lytic cycle. TGME49_282230 and TGME49_207250 were detected in the oocyst proteome and showed low transcription levels in tachyzoite and a dispensable phenotype, suggesting that these genes are only active in this stage. The subcellular localization of the proteins encoded by the ST FA genes showed a bias towards the nucleus and endoplasmic reticulum (Figure 4C). However, no clear association among them could be predicted.

There was only one FA gene (TGME49_237700, ADP/ATP carrier protein) that did not show evidence of expression either by transcriptomics or mass spectrometry (Table S3). The ADP/ATP carrier is a predicted gene that would express a peptide of 61 amino acids. We consider that more studies are required to confirm its identity as a gene.

Fifty-nine hypothetical genes (HP) were located in *T. gondii* STs, and two are compatible with FamB-like genes (Table S4). Only four show evidence of being expressed as a protein by mass spectrometry and/or LOPIT. As expected for a subtelomeric region, most of the HP genes were silenced. In those where there is evidence of expression, transcription presents a low percentile level (<50; Figure 4D). Interestingly, 8.5% of ST HP genes presented significantly differential expression levels between strains (<>50), suggesting that these genes could be associated with their phenotypic differences.

The genes of FamB and FamC families and their deduced protein products shared some aspects: they encoded low molecular weight proteins, and most of their members possessed a signal peptide and one transmembrane domain (Table 2). Members of FamB and FamC were detected in *N. caninum*, *H. hammondi*, and *T. gondii* but not in other Apicomplexa (data not shown). Analysis of bioproject PRJNA597814 [26] did not retrieve any putative FamB or FamC gene different from those already detected.

To detect putative domains and/or proteins with structural similarity, several databases and programs were searched: NCBI CDD search, Jpred 4b PDB, Motifscan, and ProtVirDB. Protein domain analysis of FamB and FamC members did not yield any significant identity rather than some short regions, mainly at the transmembrane motif (data not shown). Therefore, accumulative data suggest identity associated with integral membrane proteins as it was annotated at ToxoDB.

**Figure 5:**
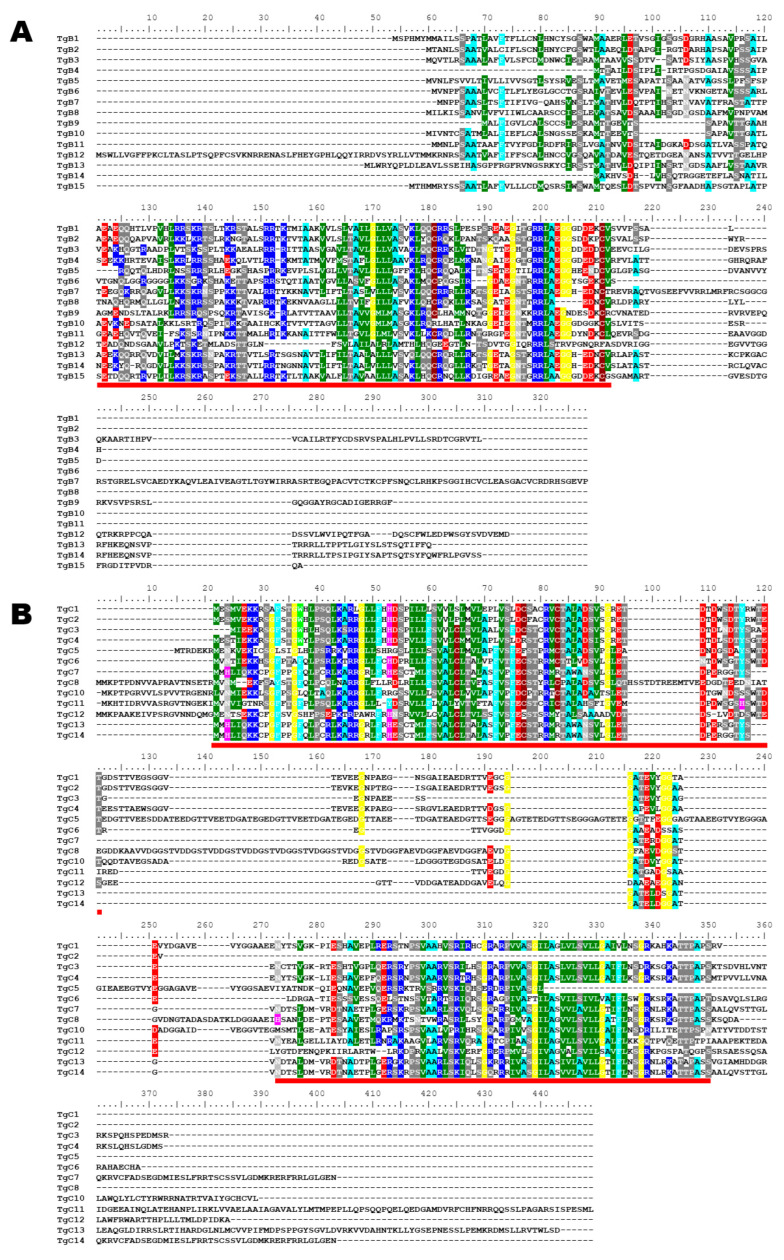
Conservation patterns of TgB and TgC families. (**A**) Alignment of amino acid sequences of TgB proteins according to Table 2 enumeration. The Red line indicates a conserved region. (**B**) Alignment of amino acid sequences of TgC proteins according to Table 2 enumeration. The red lines indicate conserved regions. Alignments were performed by MUSCLE.

The deduced amino acid sequences of the FamB and FamC proteins were aligned separately. FamB protein sequences present only one conserved region, whereas FamC has at least two conserved regions (Figure 5A,B). Both proteins present a highly variable C-terminal end. These data suggest that both FamB and FamC families encode highly variable membrane proteins.

Notably, the numbers of FamB and FamC genes annotated in the *N. caninum*, *H. hammondi*, and *T. gondii* databases are variable. For FamB, four genes were detected in *N. caninum*, seven in *H. hammondi*, and 15 in *T. gondii* (Table 2). For FamC, three genes were detected in *N. caninum*, four in *H. hammondi*, and 13 in *T. gondii* (Table 2). This observation could be indicative of an expansion of these two families in *T. gondii*.

### 2.6. Role of ST Genes in Adaptability to Different Environmental/Metabolic Situations

**Figure 6:**
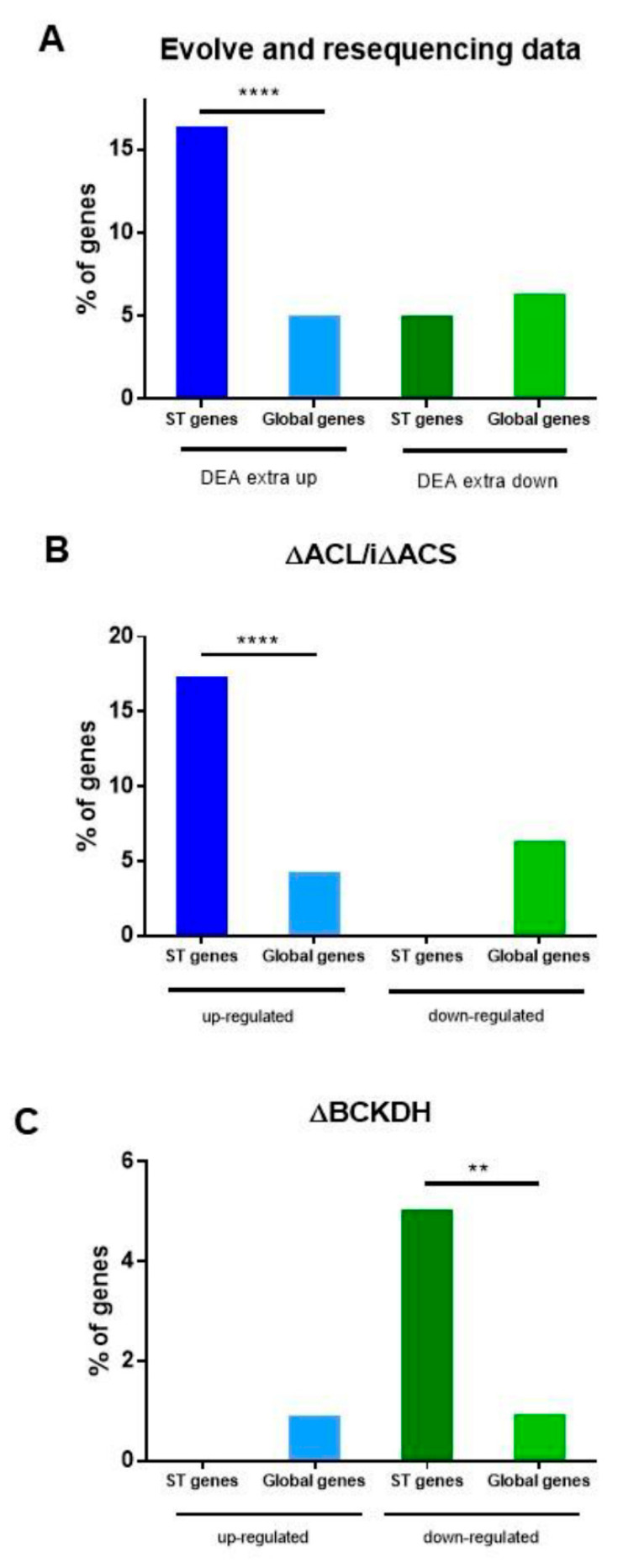
The role of ST genes in *T. gondii* environmental adaptation. (**A**) Data from a differential expression analysis (DEA) of the genes in the context of an “evolve and resequencing” study was analyzed. The ST % of genes was calculated as the number of ST genes that were upregulated (16) and downregulated (5) in DEA extra up or DEA extra down transcriptome list of the total ST genes (97). The global % of genes was calculated as the number of DEA extra up (435) and DEA extra down (551) genes from DEA extra up or extra down lists of the total GT1 strain gene data obtained from ToxoDB (8468). DEA data was obtained from Primo et al. [34] Extra: the prolonged extracellular passage of *T. gondii* tachyzoites grown in vitro. (**B**,**C**) Data from a study on the role of subcellular pools of acetyl-CoA was analyzed. (**B**) The ST % of genes was calculated as the number of ST genes that were up- (17) and downregulated (0) in the ΔACL/iΔACS mutant differentially expressed transcriptome list of the total ST genes (97). The global % of genes was calculated as the number of upregulated (376) or downregulated (75) genes from a differentially expressed transcriptome list of the total GT1 strain gene data obtained from ToxoDB (8468). ΔACL/iΔACS mutant is deficient in cytosolic/nuclear acetyl-CoA production [35]. (**C**) The ST % of genes was calculated as the number of ST genes that were up- (0) and downregulated (five) in the ΔBCKDH mutant differentially expressed transcriptome list of the total ST genes (97). The global % of genes was calculated as the number of upregulated (84) or downregulated (89) genes from a differentially expressed transcriptome list of the total Me49 strain gene data obtained from ToxoDB (8920). BCKDH mutant is deficient in mitochondrial acetyl-CoA production [35]. Data were obtained from Table S5. Fisher’s exact test was applied only to those cases in which ST genes were detected in the experiment. ** *p* < 0.01, **** *p* < 0.0001.

Our hypothesis is that ST genes contribute to adaptation to different environmental situations. Recently Primo et al. [34] analyzed the transcriptional reprogramming of *T. gondii* tachyzoites grown in vitro for 1500 generations as part of an “evolve and resequencing” experiment. In vitro, tachyzoites remain outside the host cell for a longer time, needing to adapt to that environmental context and having to increase their capacity and efficiency to enter the host cell. In this environmental context, the authors observed important transcriptional changes in genes with the transcriptional signature of extracellular parasites. We analyzed the database of differential expression analysis (DEA) of extracellular-associated genes in STs (Table S5). We detected upregulation of 16 internal ST genes out of 435 and downregulation of three border ST genes out of 551 DEA enlisted genes. Taking into account the 97 ST genes, the 16 subtelomeric genes that were upregulated represent 16.5% of the total ST genes, a significantly higher value than that represented by upregulated genes (435) in the entire genome (8468) of the GT1 strain (Figure 6A). By contrast, ST genes were poorly represented in the group of downregulated genes. Interestingly, at least two *tgc* genes and at least one *tgb* gene were upregulated, whereas euchromatin multigene FamA, FamD, and FamE genes were downregulated (Table S5).

One of the environmental conditions that can have a great impact on the growth and establishment of *T. gondii* in the host is associated with the availability of nutrients in the different tissues invaded [40]. Recently, Kloehn et al. [35] analyzed the role of subcellular sub-pools of acetyl-CoA. One of the *T. gondii* lines (ΔACL/iΔACS) presented impaired nuclear-cytosolic acetyl-CoA production due to a double mutation: ATP citrate lyase (ACL) gene knockout and acetyl-CoA synthetase inducible (i) knockdown. This could be considered a situation that mimics glucose deprivation. The transcriptome of the ΔACL/iΔACS line shows the upregulation of 376 genes and the downregulation of 75 genes. Analyzing these data, we observed that 17 ST genes (13 internal and four border genes) fall into the upregulated group, while no ST genes fall into the downregulated group (Table S5). Among them are FamB, FamC, and hypothetical genes. ST genes (17.5%) are strongly overrepresented in comparison to the group of genes (4.4%) that are upregulated in the absence of acetyl-CoA (Figure 6B). Four hypothetical genes are shared in both experiments, and none of them presented transcription evidence in normal growth conditions (Table S5). No ST gene was detected in the upregulated transcriptome of tachyzoites deficient in mitochondrion acetyl-CoA, based on *T. gondii* lacking branched-chain α-keto acid dehydrogenase-complex (BCKDH) [35]. By contrast, five ST genes (two FamB, one FamC, one HP, and one FA) were detected in downregulated genes (Table S5, Figure 6C). One of them encodes for enoyl-CoA, which is important in the metabolism of unsaturated fatty acids in beta-oxidation.

These data suggest that subtelomeric genes could play an important role in the adaptation of *T. gondii* to diverse environmental shifts, but in each case, responding with different genes.

## 3. Discussion

STs are regions of the chromosome involved in genomic integrity, the timing of DNA replication, and the transition region between euchromatin and heterochromatin. From the species point of view, STs provide a region of rapid evolution of subtelomeric genes, allowing greater plasticity and adaptability to the changing environment. In pathogens, such as *Plasmodium* spp and *Trypanosoma* spp, they are also a source of genetic diversity that contribute to pathogenicity and virulence. *T. gondii* is an example of adaptability to different environments and hosts, where STs could contribute to the species’ spread in nature. In this work, *T. gondii* STs were predicted for each chromosome based on gene density. We could define 26 STs ranging from 8.1 to 232.4 kbp, averaging 76.3 kbp. In our previous work [28], STs were defined through a comparative genomic analysis strategy. However, STs are rapidly evolving regions, and such analysis could have been partial. In fact, only nine out of 26 possible STs were detected in that study [28]. In yeast, STs were detected as low gene density regions compared to the uniform gene distribution regions that are not considered subtelomeric [15]. In the same analysis, the yeast STs were defined as the 33 kbp region from telomeres. Here, the length of telomeres was more variable. Similarly, the length of the subtelomeric regions in *T. cruzi* ranges from 5 to 182 kbp, and the authors suggested that the shortest STs could have been generated by a chromosomal break and a telomere healing event [41]. This could also explain the differences in the length of the *T. gondii* STs.

After defining the STs, we analyzed their chromatin architecture. Our detailed analysis of H2A.X, H3.3, and H3K9me3 enrichment throughout the subtelomeric region supports the heterochromatic composition of STs, as previously observed [31]. H3K9me3 is a recognized mark of repeat-rich constitutive heterochromatin [42]. However, this mark showed homogenous spreading on STs, whereas H2A.X and H3.3 were detected predominantly near telomeres and in poorly expressed or silent subtelomeric genes. H2A.X is phosphorylated at the SQE C-terminal motif under double-strand break damage, and this modified histone is called γH2A.X [43]. In oncogenic mammalian cells, H2A.X and γH2A.X were observed in STs, participating in the protection of genomic instability that can occur at the origins of replication at transcription sites [44]. The H2A.X mark was accompanied by the H3.3 mark in the *T. gondii* STs. In *Plasmodium*, H3.3 demarcates subtelomeric repeat DNA elements and GC-rich coding sequences [45]. Histone H3.3 is required for the establishment of heterochromatin during the epigenetic reprogramming of the fertilized oocyte in mammals [46]. Recently, it was observed that the phosphorylation of H3.3 in the telomeric regions/STs would modulate the state of chromatin condensation during the cell cycle and the loss of phosphorylation accompanied genomic instability and the presence of high levels of γH2A.X [47]. The pattern observed for H2A.X and H3.3 in the STs of the *T. gondii* chromosomes suggests their important role in gene silencing and establishing the heterochromatin of this region, maybe contributing to the maintenance of genome stability and TPE.

Active genes are enriched with the histone H3K4me3 and the dimer H2A.Z/H2B.Z at the 5′ end. Few cases of the sole presence of H2A.Z and/or H2B.Z in low-expression genes were observed. The presence of H3K4me3/H2A.Z/H2B.Z in the TSS region and 5′ end of active genes in *T. gondii* tachyzoites was recently observed, while the sole presence of H2A.Z/H2B.Z could be an indicator of a bivalent chromatin state [31]. In the *T. gondii* STs, a similar behavior for these histones was observed, suggesting that there is not a high level of bivalent genes.

Repetitive tandem DNA, compatible with satellite DNA, was observed in most STs but without strictly following the order of TARE1 to three, as previously described [28]. The fact that in that work, the identification of ST used the identity of end chromosomes could be the reason for this difference. The most consistent repeating element of satellite DNA corresponds to the sat350 family, followed by sat240. The rest of the satellite DNAs did not show similarities among them. While satellite DNAs are fairly homogeneous and sequence-level differences are seen between species, the high heterogeneity of satellite DNA in *T. gondii* STs is intriguing. In fact, sat350 DNA consists of highly variable units or monomers [48]. Sat350 also showed variability in numbers between strains and, very interestingly, also in the number of copies in the F1 progeny with respect to their parents [27]. Taken together, the data suggest a highly dynamic genomic region, maybe contributing to the plasticity and evolution of STs. A synteny analysis of STs on the basis of gene loss or gain, as well as gene duplication events, suggested that internal subtelomeric genes are under a highly dynamic process between strains, and this plasticity is greater when satellite DNA is present. The analysis could be biased because many STs that do not present satellite DNAs have only one gene, most of them located at the ST border. However, rather than indicating a bias, it could support the hypothesis that the lack of satellite DNA in ST is associated with lower mobilization of genes from different regions of the genome to satellite-free STs, which would argue in favor of less dynamic regions.

In addition to plasticity, the presence of satellite DNA in ST may have other roles. We observed that there are few genes overlapping with satellite DNA sequences. However, many genes are found between blocks of satellite DNAs, in some cases closely neighboring. The presence of satellite DNA has been shown to influence the expression of neighboring genes in other species [49]; therefore, some ST genes could be affected by the different ST satellite DNAs. In that sense, the association of sat240 and sat350 elements with FamC genes is intriguing. Future studies should shed light on the nature of this association.

Although the presence of long blocks of sat240 and sa350 in euchromatin was not observed, in the case of sat240, there seems to be a wide distribution of smaller short fragments, less than 100–200 bp, in almost all the chromosomes. This tandem repeat was identified in this work; therefore, there is no previous data. In contrast, the chromatin and miRNA expression of sat350 and sat529 have been determined [30]. In the future, a similar study should be carried out to infer the role of sat240 in ST but also extend to the whole *T. gondii* genome.

*Toxoplasma* STs encompass one or more genes in their territories. A total of 97 genes could be linked to STs, a large number associated with inactive chromatin and low or no transcription evidence, as expected for genes under TPE. Seventy-one of these genes encode for HP or FA proteins. Although genes linked to metabolic pathways were observed in STs [22,50], a detailed analysis indicates that there is no obvious relationship between genes located in *T. gondii* STs, with the exception of the FamB and FamC multigenic families, whose roles have not yet been identified.

Our hypothesis that ST genes would be associated with environmental stress was challenged on the basis of transcriptomes from two recent publications [34,35]. We were able to observe that, indeed, around 16 ST genes were transcriptionally associated with an adaptation to the extracellular passage, an event that may be linked to increased virulence and invasive capacity of the host [34]. Notably, no genes from the other non-subtelomeric multigene families FamA, FamD, and FamE were identified on the upregulated list, whereas they were on the downregulated list. These data suggest that FamB/C and FamA/D/E families may acquire opposite roles in the face of some environmental changes. Future studies are needed to determine if the upregulation of FamC and FamB genes is associated with invasion, host cell attachment, and extracellular stress.

Similarly, 17 ST genes were shown to be associated with cytosolic acetyl-CoA deficiency. Cytosolic acetyl-CoA is a major metabolite of the tricarboxylic acid pathway and energy production. It also regulates the acetylation of several proteins, and its lack in *T.gondii* showed a deficiency in the acetylation of several proteins, mainly histones H2A.Z, H2B.Z, H2Bb, and H4 [35]. This aspect would link the role of histone acetylation as sensors of nutrient changes in the environment with the activation of genes that adapt to nutritional stress [51,52], where the ST genes would have a relevant role given their enrichment. The transcriptome of the BCKDH line, which produces acetyl-CoA in the mitochondrion, also includes a small group of ST genes, only in the case of those that are downregulated. The genes of the FamB and FamC family (three out of five) are also highly enriched. One of downregulated ST genes is enoyl-CoA, associated with the beta-oxidation of fatty acids, a process that leads to the formation of acetyl-CoA. Loss of BCKDH expression also leads to hypoacetylation of mitochondrial proteins. Clearly, ST genes present a complex regulation mechanism under different metabolic stresses. Future studies should shed light on the role of these genes.

## 4. Materials and Methods

### 4.1. Sequence Analysis

FamB and FamC orthologues were retrieved from the NCBI database, www.toxoDB.org, www.plasmodb.org, www.cryptodb.org, and www.piroplasmadb.org (accessed on 6 March 2022), using a Blastp search. Every *T. gondii* FamC and FamB protein sequence was used as query sequences. The signal peptide probability was confirmed by SignalP 5.0 (https://services.healthtech.dtu.dk/service.php?SignalP-5.0, accessed on 15 March 2022). The signal peptide cleavage site was predicted by PSORT II (https://psort.hgc.jp/form2.html, accessed on 15 March 2022). The TM region was retrieved from PSORT II. The signal peptide and transmembrane domains were confirmed from the ToxoDB report. The repetitive DNA regions were determined by a dot plot analysis on the Yass webpage (https://bioinfo.lifl.fr/yass/yass.php, accessed on 28 August 2022) [53]. The analysis was extended to the first 100 kbp of each chromosome end, except when the border of the proposed ST extended beyond 100 kbp. The identity of the DNA satellites was confirmed using a combination of the Yass and Blastn programs (https://blast.ncbi.nlm.nih.gov/, accessed on 13 May 2022). The gene density analyses were performed in the ToxoDB using a manual scan of the genome Browser, versions 53 and 57. Additionally, the synteny of the genes detected in Me49 was determined in the 14 strains, including Me49 in ToxoDB (version 53), by manual inspection, orthology, and synteny section.

Alignments were performed by the MUSCLE program (Molecular Evolutionary Genetics Analysis, MEGA-X) at standard conditions: Gap open: −2.9, Gap extend: 0, Hydrophobicity multiplier: 1.2, Max memory in MB: 2018, Max interactions: 16, Cluster methods: UPGMA, and Min diag length (lambda): 24. FamB and FamC predicted proteins were searched by Motif scan (https://myhits.sib.swiss/cgi-bin/motif_scan, accessed on 5 April 2022); SMART (http://smart.embl-heidelberg.de/, accessed on 5 April 2022); NCBI Conserved Domains (https://www.ncbi.nlm.nih.gov/Structure/cdd/wrpsb.cgi, accessed on 5 April 2022); J-Pred (http://www.compbio.dundee.ac.uk/jpred/, accessed on 5 April 2022) and ProtVirDB (http://bioinfo.icgeb.res.in/protvirdb, accessed on 23 April 2022) software. New data on the *N. caninum* genome was generated and available at https://www.ncbi.nlm.nih.gov/bioproject/, accessed on 15 January 2022); under accession number PRJNA597814. Since genes are still not annotated, the presence of additional putative FamB and FamC genes was searched using tblast.

### 4.2. Chromatin Analysis

ChIP-seq raw data publicly available was downloaded from GSE98806 and GSE104347 (GEO-NCBI). The specific samples used for this study are summarized in Table S1. Paired- or single-end reads were aligned against the *T. gondii* Me49 genome version 53 using Bowtie 2 [54]. Genome-wide coverage profiles were computed in R (Bioconductor GenomicRanges) and normalized so that the average coverage for every chromosome was 1. The normalized coverage profiles were saved as bigwig files and visualized in IGV [55]. ChIP-seq peaks for histone marks were called with MACS2 [56,57] using a threshold for the q-value of 0.05. The occupancy peaks called by MACS2 were assessed manually in the Integrative Genomics Viewer (IGV, Broad Institute) by comparing them with the coverage profiles computed in R.

For heatmaps representations of the 97 annotated ST genes (Table S1), genome-wide coverage profiles were represented relative to the 5′ end using Deeptools (https://deeptools.readthedocs.io/en/develop/, accessed on 25 May 2022) [58]. The genomic location for the represented group of genes was obtained from ToxoDB. The 5′ end corresponds to the 5′ untranslated region (UTR) when annotated or to the first ATG of the coding sequence (CDS). For correlation with transcription levels, previously quantified RNA-seq data was used by Crocken et al. [59]. The average of 3 biological replicates grown and harvested at pH 7 was used for gene sorting.

### 4.3. Statistical Analysis

Statistical analysis was performed using GraphPad Prism 6.0 software (GraphPad Software Inc., San Diego, CA, USA). The results were analyzed using Fisher’s exact test. *p*-values < 0.05 were considered to be statistically significant.

## 5. Conclusions

Here, we have generated a more precise idea of ST chromatin’s architecture, plasticity, and organization. The presence of 97 ST genes is highlighted. The preliminary characterization of these genes shows differences in expression and degrees of interstrain conservation with properties that could be consistent with virulence- and/or adaptability-associated genes. In addition, the lack of conservation of some STs genes added to their overrepresentation and upregulation as a consequence of metabolic shift or a non-normal extracellular passage would indicate that STs genes could be involved in the adaptability of *T. gondii* to different environments. Among the subtelomeric genes, there are two multigene families (FamB and FamC) of putative integral membrane proteins with high variable C-terminal region that seemed to be expanded in *T. gondii* compared with *N. caninum* and *H. hammondi*. Their role remains intriguing and encourages further study to determine their relevance in *T. gondii* biology and pathogenicity. Some of them are regulated by environmental or metabolism shifts. The rapid evolution rate and diversity of FamB and FamC genes may contribute to *T. gondii’s* adaptation to different hosts or host cell types favoring its expansion. In addition to ST genes, we also found several examples of satellite DNA, among them the already characterized sat350 and a new element called sat240. It is important to highlight that satellite DNAs have been shown to generate a wide variety of non-coding RNAs [30,60]. Although this study was not addressed in this work, Braun et al., 2010 [30] showed that numerous miRNAs cover sat350 and sat529 sequences. Future studies should also analyze the possible transcription of ST lncRNAs.

## Figures and Tables

**Table 1:** List and main features of *T. gondii* STs.

ST	Length (kpb)	Nr of Genes	CG% ^1^	Telomere Sequences	Satellite DNA
ST_Ia_L	15.6	1	50.1	no	yes
ST_Ia_R	10.5	2	49.0	yes	no
ST_Ib_L	18.4	3	52.0	yes	no
ST_Ib_R	40	2	53.3	yes	yes
ST_II_L	22.2	1	49.7	no	no
ST_II_R	109.4	4	53.3	yes *	yes
ST_III_L	35.7	1	53.3	yes	no
ST_III_R	232.4	5	52.5	yes	yes
ST_IV_L	77	5	54.8	yes **	yes
ST_IV_R	40.2	4	50.8	no	yes
ST_V_L	112.6	5	54.3	no	yes
ST_V_R	177.9	5	54.4	no	yes
ST_VI_L	8.1	1	50.1	yes	no
ST_VI_R	53.7	7	52.7	no	yes
ST_VIIa_L	58.4	6	52.6	yes	yes
ST_VIIa_R	76.3	1	53.7	yes	no
ST_VIIb_L ^+^	82.9	7	53.4	yes	yes
ST_VIII_R ^+^	53.1	1	54.9	yes	yes
ST_IX_L	179.1	4	53.9	no	yes
ST_IX_R	49.6	8	52.2	yes	no
ST_X_L	139.8	5	52.3	no	yes
ST_X_R	27.5	3	50.1	no	yes
ST_XI_L	88.2	7	51.4	yes	yes
ST_XI_R	13.2	1	53.1	yes	no
ST_XII_L	47.6	3	51.6	no	yes
ST_XII_R	215	4	52.3	yes	yes
average	76.3	3.7	52.37	-	-
sum	1985.4	96	-	16 (yes)	18 (yes)
Me49 Chr ^++^	54,997.637	8920	-	-	-

^+^ Chromosomes VIIb and VIII turned out to be fused according to recent alignments, not yet annotated. Here the left arm for VIIb and the right arm for VIII were taken into account. ^++^ Data obtained from ToxoDB, organism Me49. ^1^ Telomere sequences and “ns” were removed from the analysis (https://www.sciencebuddies.org/science-fair-projects/references/genomics-g-c-content-calculator, accessed on 11 May 2022). *Toxoplasma* GC content 52% (RH strain). * Interstitial, position 1445–1758. ** Interstitial, position 1896–2705.

**Table 2:** Basic gene and protein features of ST genes with annotated description.

Gene	geneID (TGME49)	Ch	Protein Length (aa)	TM	SP Score	Phenotype ^1^	Transcription ^1,§^ Me49/RH	Proteomic ^1^	LOPIT ^1^
*T. gondii* FA ^2^:									
chapA-tub	_295310	I ^a^	189	0	<0.01	−4.78	85.7/85.6	yes	nucleolus
ts	_220840	II	571	0	<0.01	1.76	20.7/7.66	Oocyst ^	NDA
otu-cp familiy	_237894	IV	219	0	<0.01	−4.37	39.9/38.8	NDA	NDA
enoyl-CoA	_317705	IV	371	0	<0.01	−0.45	93/92.3	yes	nucleolus
eIF3-D	_317720	IV	609	0	<0.01	−4.87	90.8/92.7	yes	nucleus
otu-cp	_237900	IV	486	0	<0.01	NDA	39.9/38.8	yes	NDA
ppi	_238000	VI	283	0	<0.01	−6.27	83.2/87.1	yes	nucleolus
adp/atp	_237700	VI	61	0	<0.01	−0.76	NDA	NDA	NDA
sa-t/k	_282230	VII ^a^	634	0	<0.01	0.94	10.2/6.1	Oocyst ^	NDA
electron-t	_207250	X	450	0	<0.01	0.73	33.4/16.6	Oocyst ^	NDA
vtc2	_298630	XI	1308	2	<0.01	−3.3	31.9/35.4	yes	ER/Golgi/PM
ER-oxired	_300380	XII	587	1	0.02	0.82	63.7/74.8	yes	ER
FamB and FamC multigene families:									
TgB1	_200480	VIII	167	1	0.19/ +	1.23	NDA	NDA	NDA
TgB2	_244920	VI	162	1	0.39	−0.82	NDA	NDA	NDA
TgB3	_264225	VIIb	208	2 ^Φ^	0.72	2.03	NDA	NDA	NDA
TgB4	_274290	IX	136	1	<0.01	0.9	91.8/87.7	NDA	NDA
TgB5	_276230	III	163	1	0.87/ +	2.38	69.9/85.4	NDA	NDA
TgB6	_279460	IX	149	1	0.82/ +	0.43	NDA	NDA	NDA
TgB7	_283450	V	265	1	0.35	1.72	67.0/25.3	NDA	NDA
TgB8	_295300	Ia	158	1	0.87/ +	0.42	60.7/24.7	NDA	NDA
TgB9	_298090	II	172	1	0.91/ +	−0.24	NDA	NDA	NDA
TgB10	_299570	IX	150	1	0.85/ +	−1.12	50.9/31.0	NDA	NDA
TgB11	_301890	X	165	1	0.012	NDA	91.0/82/2	NDA	NDA
TgB12	_307045	XI	258	1	0.17/ +	1.30	63.7/37.9	NDA	NDA
TgB13	_307460	V	212	1	<0.01	1.16	NDA	NDA	NDA
TgB14	_307480	V	177	1	<0.01	0.28	NDA	NDA	NDA
TgB15	_320770	IV	182	1	0.89/ +	−0.05	NDA	NDA	NDA
HhB3	_264225	NA	264	2 ^Φ^	0.064	NDA	NDA	NDA	NDA
HhB6	_279460	NA	152	1	0.84/ +	NDA	NDA	NDA	NDA
HhB7	_283450	NA	126	1	<0.01	NDA	NDA	NDA	NDA
HhB8	_295300	NA	158	1	0.92/ +	NDA	NDA	NDA	NDA
HhB9	_298090	NA	137	0	0.92/ +	NDA	NDA	NDA	NDA
HhB10	_299570	NA	107	1	0.91/ +	NDA	NDA	NDA	NDA
HhB16	_454170	NA	183	1	0.79/ +	NDA	NDA	NDA	NDA
NcB13	CEL64573 *	Ib ^a^	163	1	0.71	NDA	NDA	NDA	NDA
NcB17	_060290	XII	147	1	<0.01	NDA	NDA	NDA	NDA
NcB18	CEL66112	NDA	199	1	0.30	NDA	NDA	NDA	NDA
NcB19	CEL64819 *	III ^b^	173	1	<0.01	NDA	NDA	NDA	NDA
TgC1	_321170	III	232	2 ^Φ^	0.13	0.52	44.7/61.2	NDA	NDA
TgC2	_200700	X	139	1 ^Φ^	0.05	NDA	NDA	NDA	NDA
TgC3	_307260	XI	206	1	0.14 +	0.96	75.2/64.8	NDA	NDA
TgC4	_200130	XII	238	1	0.04 +	NDA	NDA	NDA	NDA
TgC5	_298960	III	282	1 ^Φ^	<0.01	0.06	53.9/40.5	NDA	NDA
TgC6	_200590	X	201	1	0.01 +	2.09	NDA	NDA	NDA
TgC7	_287280	Ia	225	1	0.06 +	NDA	53.0/36.9	NDA	NDA
TgC8	_220080	V	306	1	<0.01	0.44	23.7/22.4	Membrane	NDA
TgC10	_228780	X	286	2	<0.01	1.49	15.2/23.9	NDA	NDA
TgC11	_298060	II	305	3 ^Φ^	<0.01	1.22	14,5/32.6	NDA	NDA
TgC12	_300990	IV	252	1	<0.01	0.04	18.4/16.0	NDA	NDA
TgC13	_292610	V	265	1	0.06 +	NDA	53.0/36.9	NDA	NDA
TgC14	_329400	NA	225	1	0.06 +	NDA	53.0/36.9	NDA	NDA
HhC6a	_321170	NA	216	1	0.02	NDA	NDA	NDA	NDA
HhC6b	_200590	NA	193	1	0.06 +	NDA	NDA	NDA	NDA
HhC8	_220080	NA	180	1^c^	0.004	NDA	NDA	NDA	NDA
HhC11	_298060	NA	330	1	<0.01	NDA	NDA	NDA	NDA
NcC15	CEL68745 *	IX	313	2 ^Φ^	0.01	NDA	NDA	NDA	NDA
NcC16	_069620	NA	242	1	0.03	NDA	NDA	NDA	NDA
NcC17	CEL66113 *	VIIa ^c^	146	1	0.005	NDA	NDA	NDA	NDA

TGME49: *Toxoplasma gondii* strain Me49. Tg: *T. gondii*; Hh: *Hammondia hammondi*; Nc: *Neospora caninum.* SP: signal peptide: Score was determined by Signal P 5.0 (https://services.healthtech.dtu.dk/service.php?SignalP-5.0, accessed on 15 March 2022). + Signal peptide predicted by PSORT II (https://psort.hgc.jp/form2.html, accessed on 15 March 2022). ER: endoplasmic reticulum; TM: transmembrane region (Psort II, TMHMM server v 2.0, http://www.cbs.dtu.dk/services/TMHMM/0, accessed on 15 March 2022). NA: not assigned; NDA: no data available. ^1^ Phenotype, transcription, proteomic, and LOPIT data were obtained from ToxoDB. ^§^ Only the transcription “expression profiling of three archetypal lineages” database was incorporated. ^2^ FA: functional annotated genes. ToxoDB annotation: chapA-tub: tubulin binding cofactor A protein; ts: threonine synthase; out-cp family: OTU family cysteine protease; enoyl-coA: enoyl-CoA hydratase/isomerase family protein; eIF3-D: eukaryotic initiation factor 3, subunit D; ppi: peptidyl-prolyl isomerase; adp/atp: adp/atp translocase; sa-t/k: sulfate adenylyltransferase/adenylylsulfate kinase; electron-t: electron-transfer-flavoprotein; vtc2: vacuolar transporter chaperone VTC2; ER-oxired: endoplasmic reticulum oxidoreductin. ^ Oocyst proteome. ^Φ^ One is a TMHH in the putative cleavage site. * Bioproject PRJEB7872. ^a^ Chromosome corresponding to gDNA sequence NCLIV_004620, a truncated version (87aa). ^b^ Chromosome corresponding to gDNA sequence NCLIV_006930, a truncated version (113aa). ^c^ Chromosome was determined by sequence NCLIV_019380, a truncated version (122aa) of CEL66113.

## Data Availability

The data that support the findings of this study are available from the corresponding author upon reasonable request.

## Supplementary Materials

The following supporting information can be downloaded at: https://www.mdpi.com/article/10.3390/epigenomes6030029/s1, Table S1: List and description of *T. gondii* STs. Table S2: Major datasets analyzed. Table S3: RNA-seq data of 97 ST genes. Table S4: List and description of ST single hypothetical genes. Table S5: List of ST genes detected in differential expression analysis (DEA). Figure S1. Chromatin landscape of the *T. gondii* subtelomeric regions. Normalized coverage profiles showing the location of the histones H3K9me3 (violet), H3.3 (yellow), and H2A.X (red) for the different subtelomeric regions proposed (green rectangle). The analyzed regions extend beyond the STs arbitrarily in each case. Below, in black, the gene location is indicated. The vertical light blue line indicates the presence of telomeric repeat. The dashed vertical blue line indicates interstitial telomeric repeat. The red ? indicates the possibility that the ST region extends beyond de defined one. Occupancies of histone marks were plotted with the Integrated Genomics Viewer (IGV). Scale: H3K9me3, 0–4.21; H3.3, 0–1.76 and H2A.X, 0–2.05. Figure S2. Satellite DNA identification in STs. The determination of the repetitive DNA regions was carried out by dot plot analysis, using 100 kbp from each end for all STs (ST) that did not exceed that length. In those STs that exceeded 100 kbp, the length was taken to the border of the ST. In the case of the STs of the left arms (L), direct sequences were used. In the cases of the STs of the right arm (R), reverse complementary (RC) sequences were used. The location of satellite DNAs sat240 and sat350 are indicated in the figure. Figure S3. Presence of sat240 and sat350 on *T. gondii* chromosomes. To search for sat240 and sat350 satellite DNA elements, a sequence block of different repeats was generated to track its presence in *T. gondii* chromosomes by dot plot. For sat240 we used the sequences of the mentioned repeat from chromosomes Ia (position 3393–4035), III (position 2,308,702- 2,310,007), V (position 103,058–105,247), X (position 64,449–66,824), XII (position 7,042,760- 7,044,628), generating a 8432-bp sequence block. For sat350 we used parts of the repeat sequences from chromosomes II (position 2,257,761–2,271,082), III (position 2,323,699–2,327,108), V (position 62,336–81,515), XII (position 4952–9238), and the GenBank sequences AY333785, AF022237, AY330209 and M57917, generating a 43,507-bp sequence block. All sequences were oriented in the same direction. Each sequence block was compared to the complete chromosome sequences. Once the dot plot was obtained, the similarity is shown as vertical lines. Green lines represent direct alignment, and red lines represent complementary reverse alignment. An interactive analysis was performed on each line to determine the length of the similar sequence. According to the analysis, the presence of sat240 was represented as light green arrowheads, whereas sat350 as dark green arrowheads. *: sequence of 240–300 bp. #: sequence of 350 bp. The rest of the vertical lines presented similar fragments of less than 200 bp. The scale varies for each chromosome. Note that when there are no significantly similar DNA fragments in the comparison, the program provides truncated versions of the chromosome. Figure S4. Arrays of genes and satellite DNA sequences present in STs. Based on the dot plot analysis and data from the Table S1, a map of the position of each gene (top) and satellite DNA (bottom) was constructed for each ST. The figure shows a selection of STs that contain satellite DNA different from sat240 or sat350. The length of satellite DNAs and genes are scaled for each ST. Note that each ST has a different scale according to its length. Satellite DNA: grey. The names of the genes respond to the criteria of the additional file Table S1. Ns: track of N (any nucleotide) in the ST sequence and genomic gene.
