# Supplementary material for: Architecture, Chromatin and Gene Organization of Toxoplasma gondii Subtelomeres
Source: Epigenomes. 2022 Sep 15;6(3):29. doi: 10.3390/epigenomes6030029 (PMC9498087; doi:10.3390/epigenomes6030029)
Supplement: Supplementary file 1 [file epigenomes-06-00029-s001.zip › FiguresSupl.pdf]

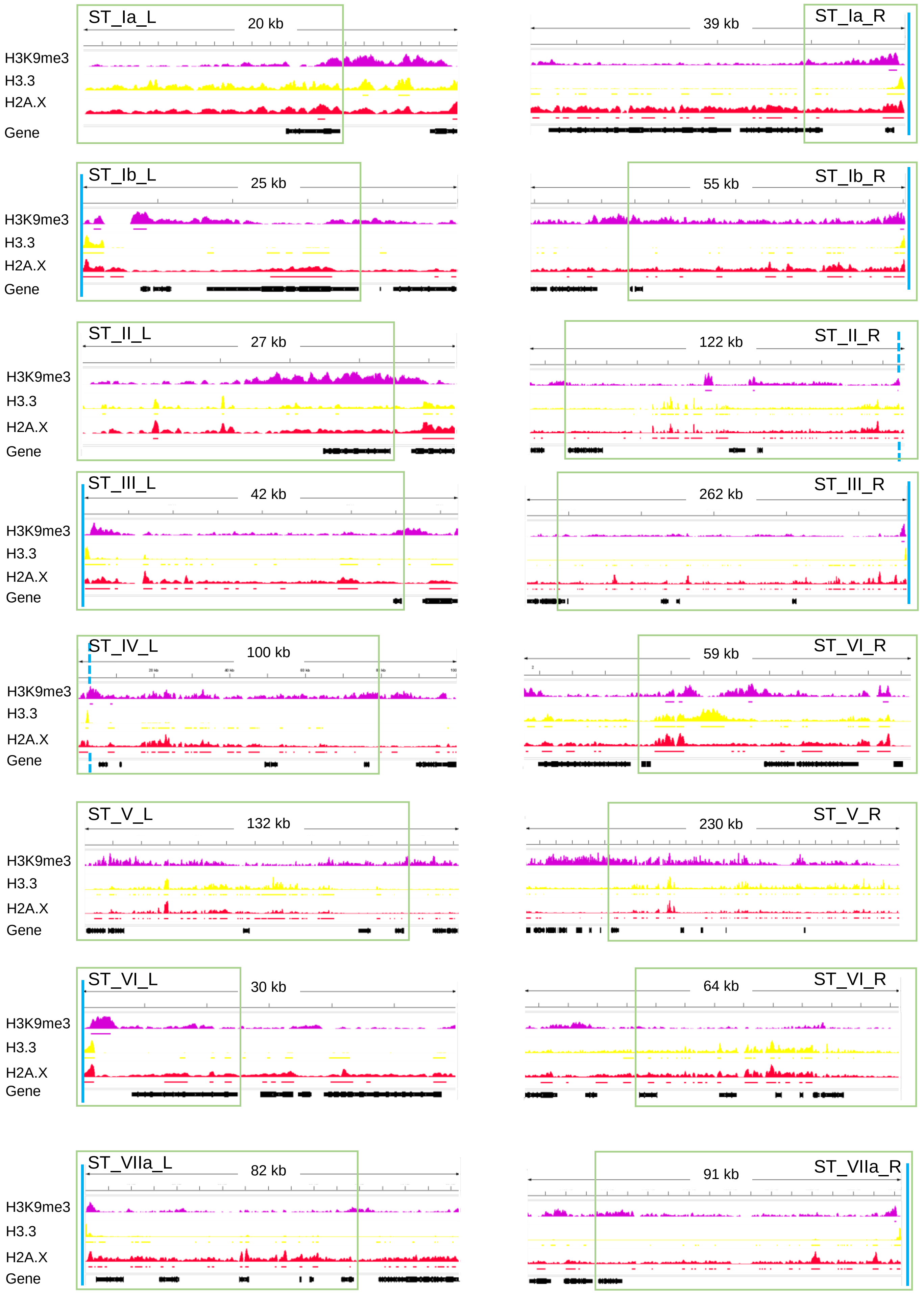

ST_Ia_L
20 kb
ST_Ia_R
39 kb
ST_Ib_L
25 kb
ST_Ib_R
55 kb
ST_II_L
27 kb
ST_II_R
122 kb
ST_III_L
42 kb
ST_III_R
262 kb
ST_IV_L
100 kb
ST_VI_R
59 kb
ST_V_L
132 kb
ST_V_R
230 kb
ST_VI_L
30 kb
ST_VI_R
64 kb
ST_VIIa_L
82 kb
ST_VIIa_R
91 kb
H3K9me3
H3.3
H2A.X
Gene

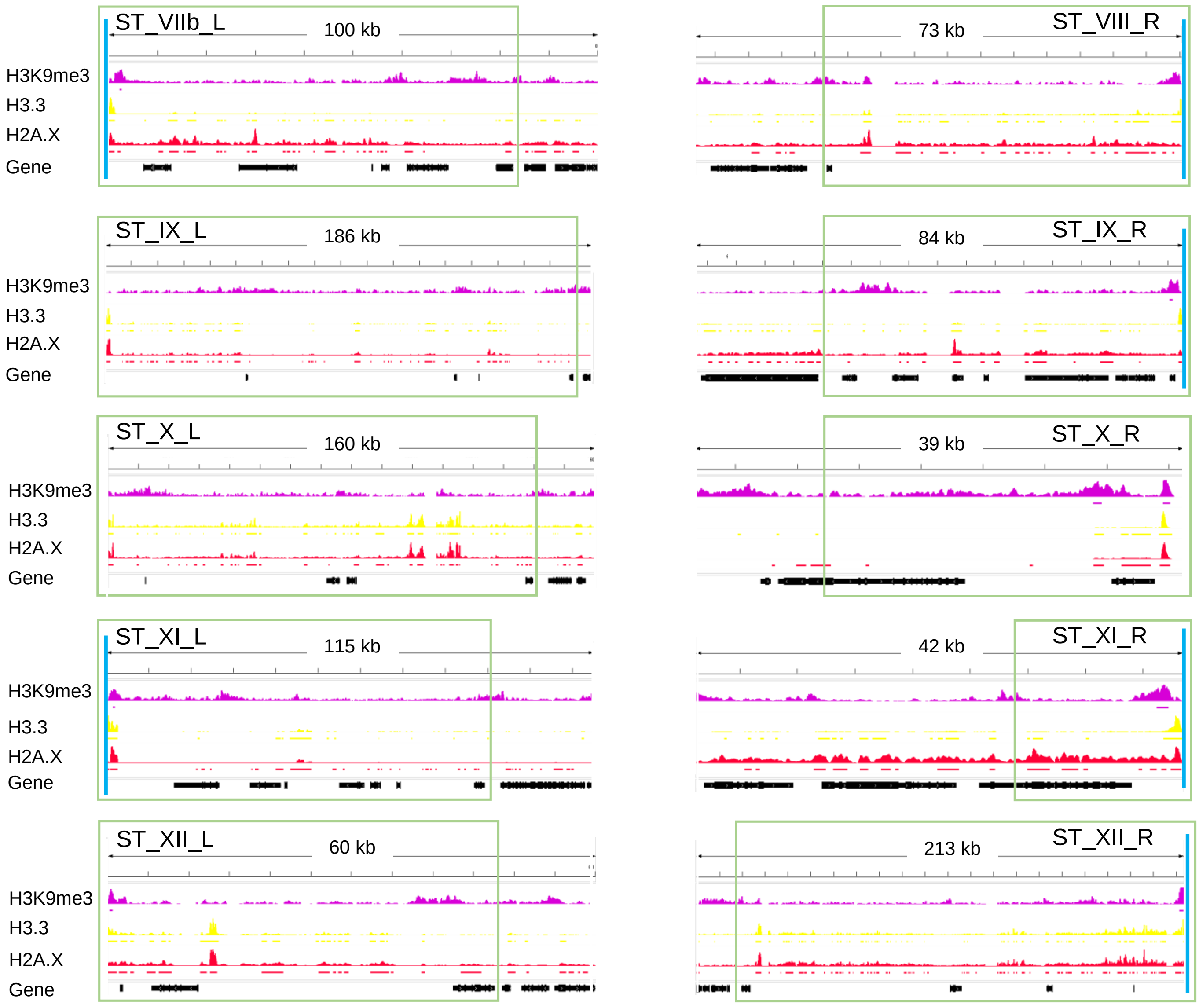
ST_VIIb_L
100 kb
ST_VIII_R
73 kb
ST_IX_L
186 kb
ST_IX_R
84 kb
ST_X_L
160 kb
ST_X_R
39 kb
ST_XI_L
115 kb
ST_XI_R
42 kb
ST_XII_L
60 kb
ST_XII_R
213 kb
H3K9me3
H3.3
H2A.X
Gene

Figure S1

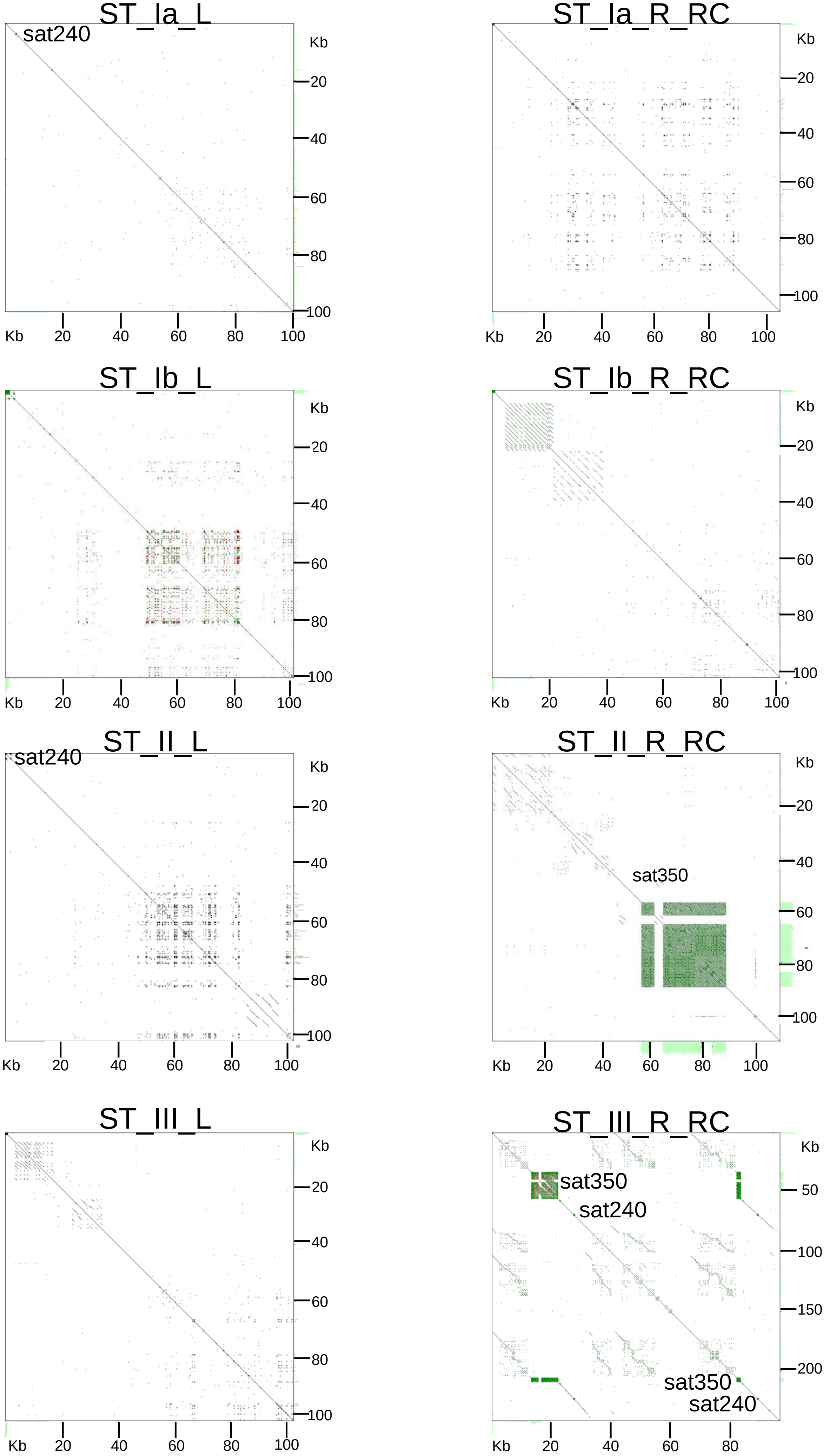
ST_Ia_L
sat240
Kb
20
40
60
80
100
Kb
20
40
60
80
100
ST_Ia_R_RC
Kb
20
40
60
80
100
Kb
20
40
60
80
100
ST_Ib_L
Kb
20
40
60
80
100
Kb
20
40
60
80
100
ST_Ib_R_RC
Kb
20
40
60
80
100
Kb
20
40
60
80
100
ST_II_L
sat240
Kb
20
40
60
80
100
Kb
20
40
60
80
100
ST_II_R_RC
sat350
Kb
20
40
60
80
100
Kb
20
40
60
80
100
ST_III_L
Kb
20
40
60
80
100
Kb
20
40
60
80
100
ST_III_R_RC
sat350
sat240
sat350
sat240
Kb
50
100
150
200
Kb
20
40
60
80

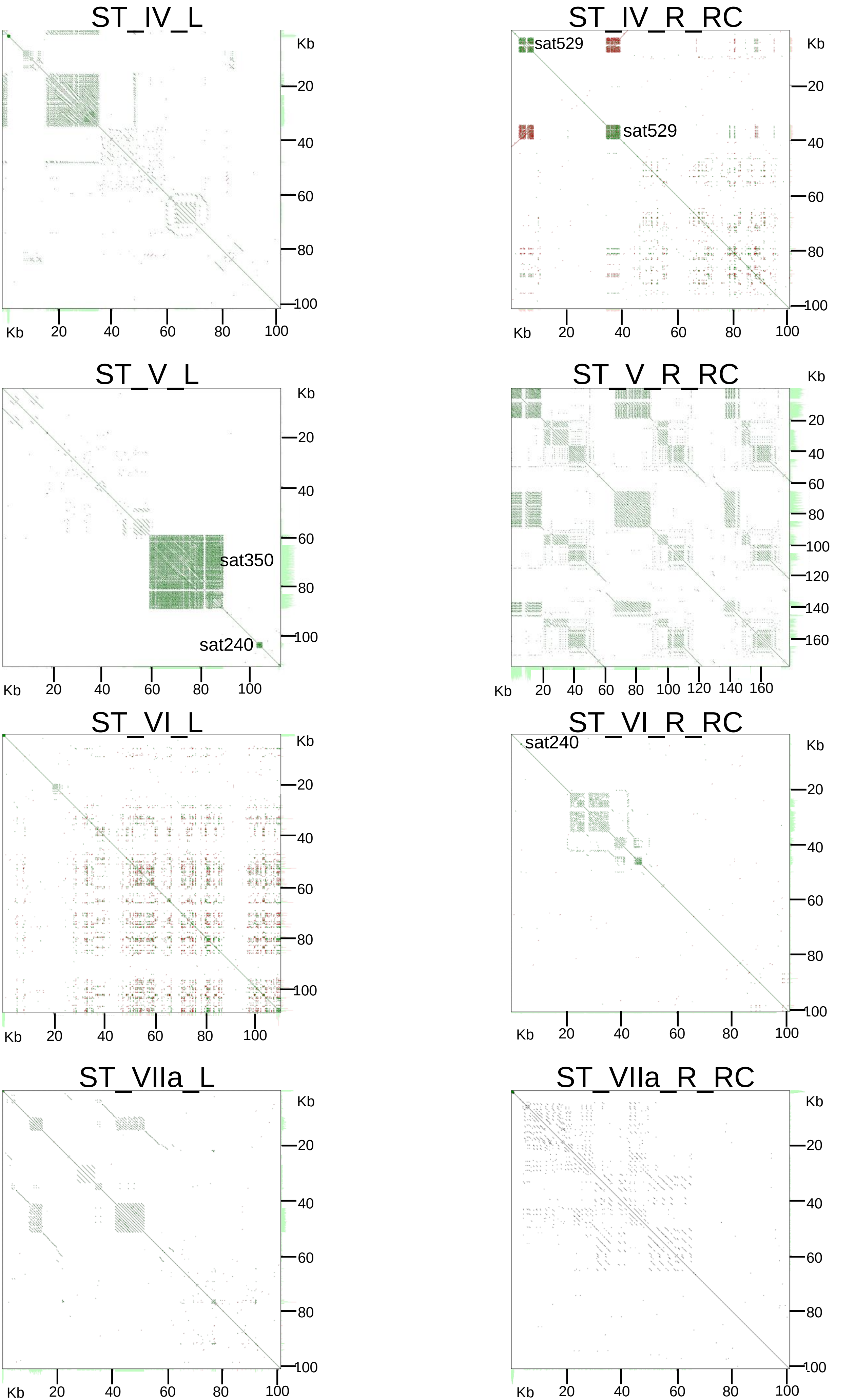
ST_IV_L
ST_IV_R_RC
sat529
sat529
ST_V_L
sat350
sat240
ST_V_R_RC
ST_VI_L
ST_VI_R_RC
sat240
ST_VIIa_L
ST_VIIa_R_RC
Kb
20
40
60
80
100
120
140
160

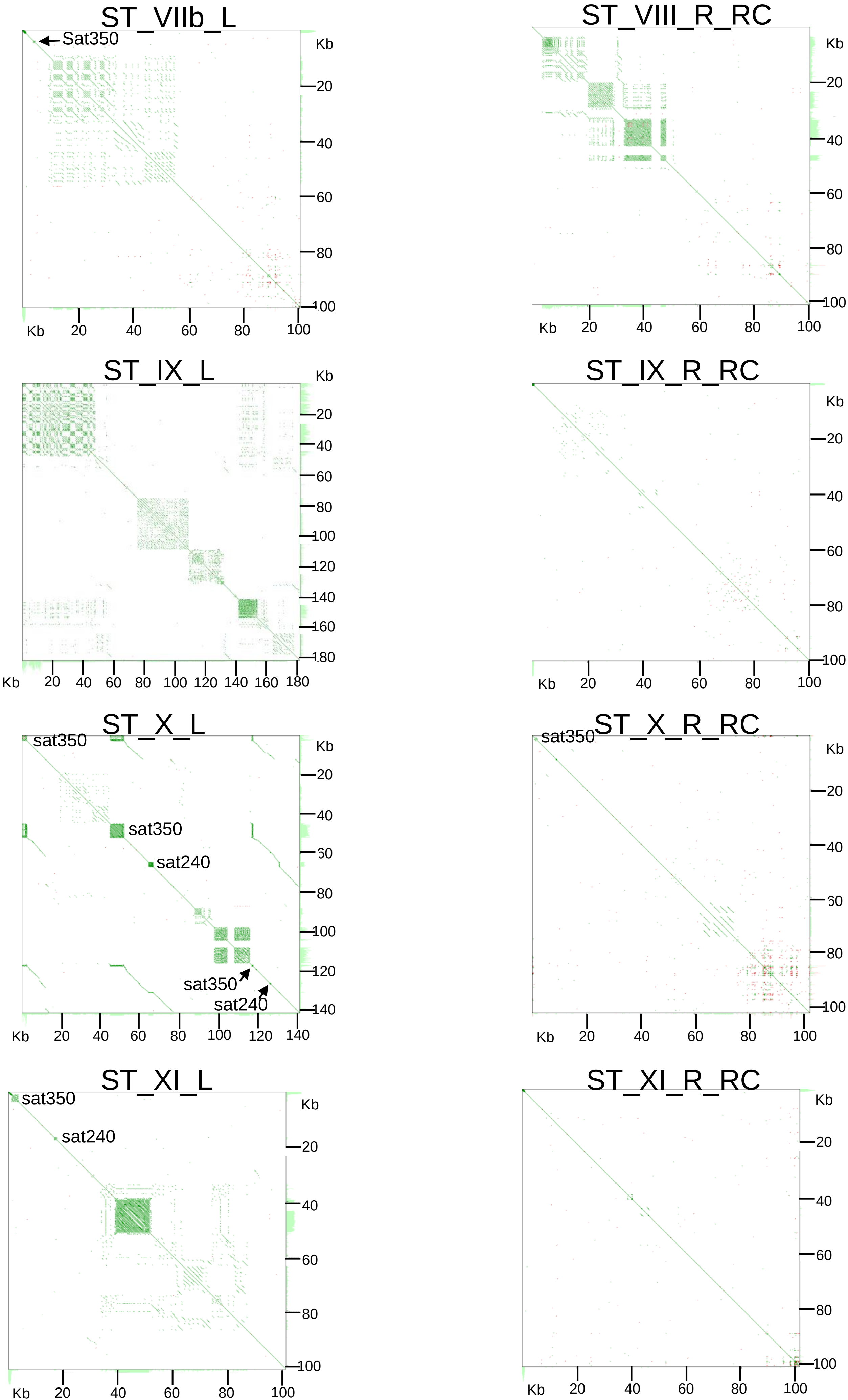

ST_VIIb_L
Sat350
Kb
20
40
60
80
100
ST_VIII_R_RC
Kb
20
40
60
80
100
ST_IX_L
Kb
20
40
60
80
100
120
140
160
180
ST_IX_R_RC
Kb
20
40
60
80
100
ST_X_L
sat350
sat350
sat240
sat350
sat240
Kb
20
40
60
80
100
120
140
ST_X_R_RC
sat350
Kb
20
40
60
80
100
ST_XI_L
sat350
sat240
Kb
20
40
60
80
100
ST_XI_R_RC
Kb
20
40
60
80
100

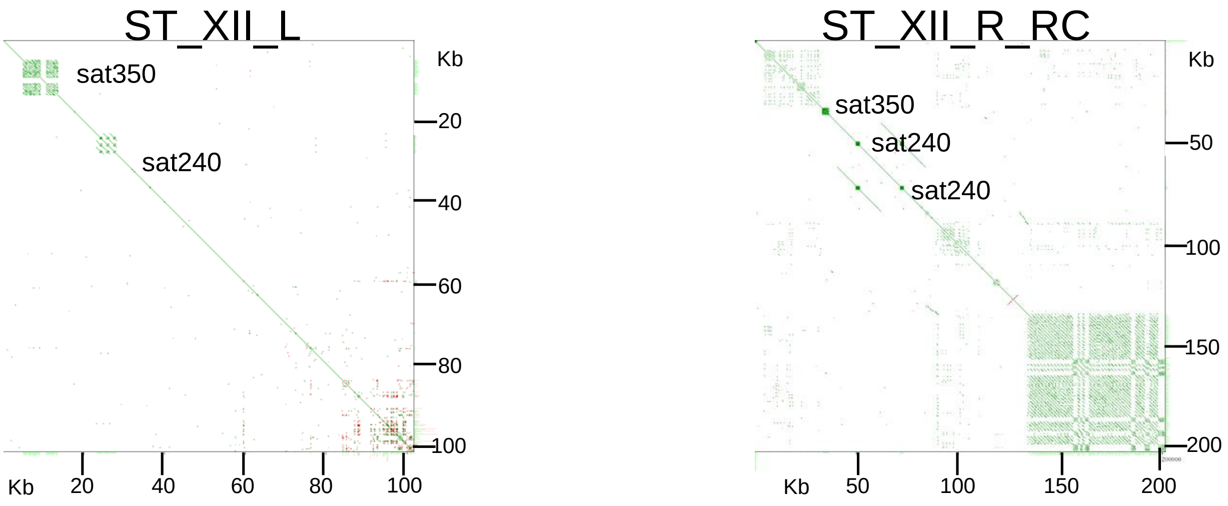
ST_XII_L
sat350
sat240
Kb
20
40
60
80
100
Kb
20
40
60
80
100
ST_XII_R_RC
sat350
sat240
sat240
Kb
50
100
150
200
Kb
50
100
150
200

Figure S2

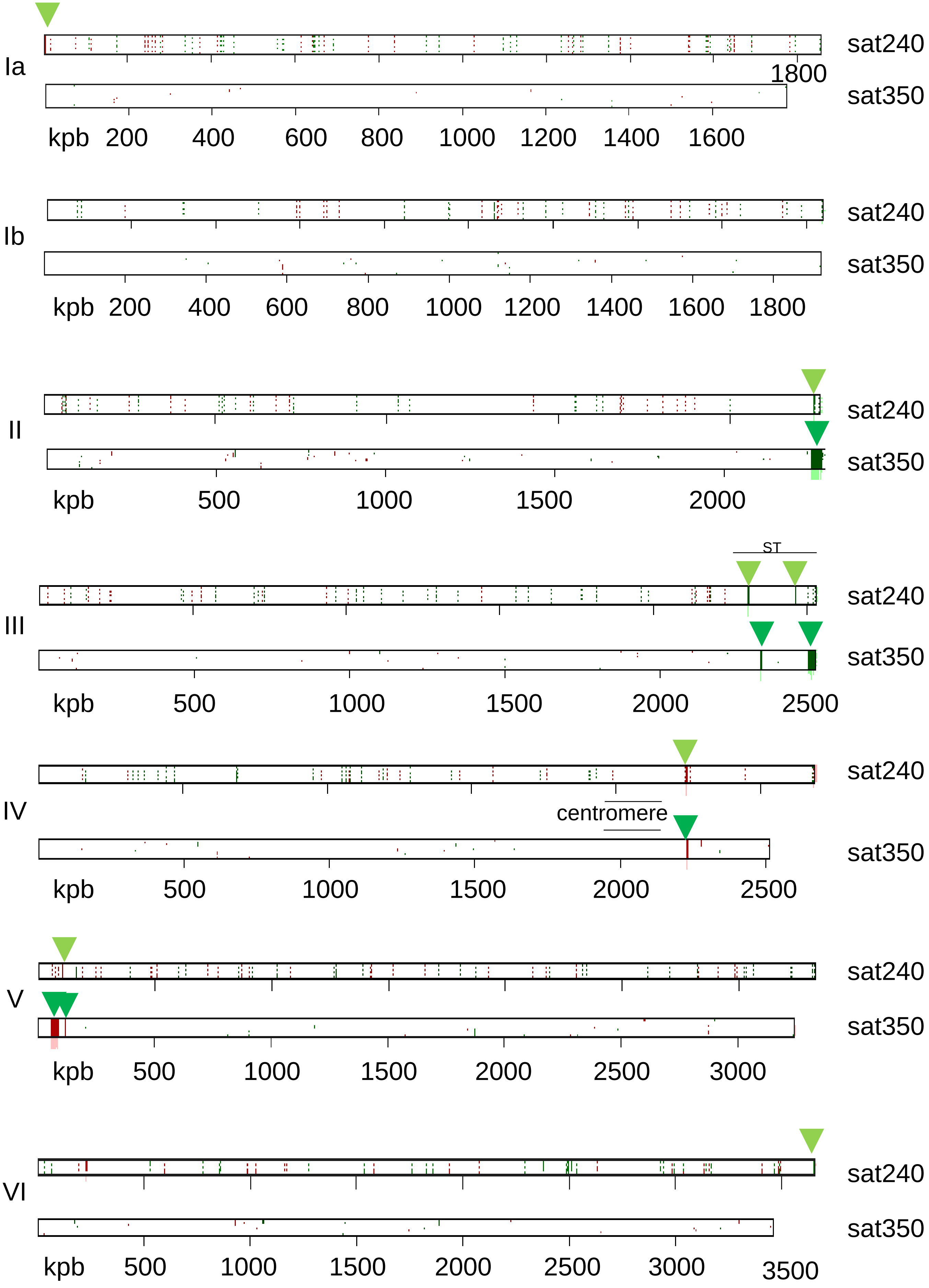
Ia
sat240
1800
sat350
kpb 200 400 600 800 1000 1200 1400 1600
Ib
sat240
sat350
kpb 200 400 600 800 1000 1200 1400 1600 1800
II
sat240
sat350
kpb 500 1000 1500 2000
ST
III
sat240
sat350
kpb 500 1000 1500 2000 2500
IV
sat240
centromere
sat350
kpb 500 1000 1500 2000 2500
V
sat240
sat350
kpb 500 1000 1500 2000 2500 3000
VI
sat240
sat350
kpb 500 1000 1500 2000 2500 3000 3500

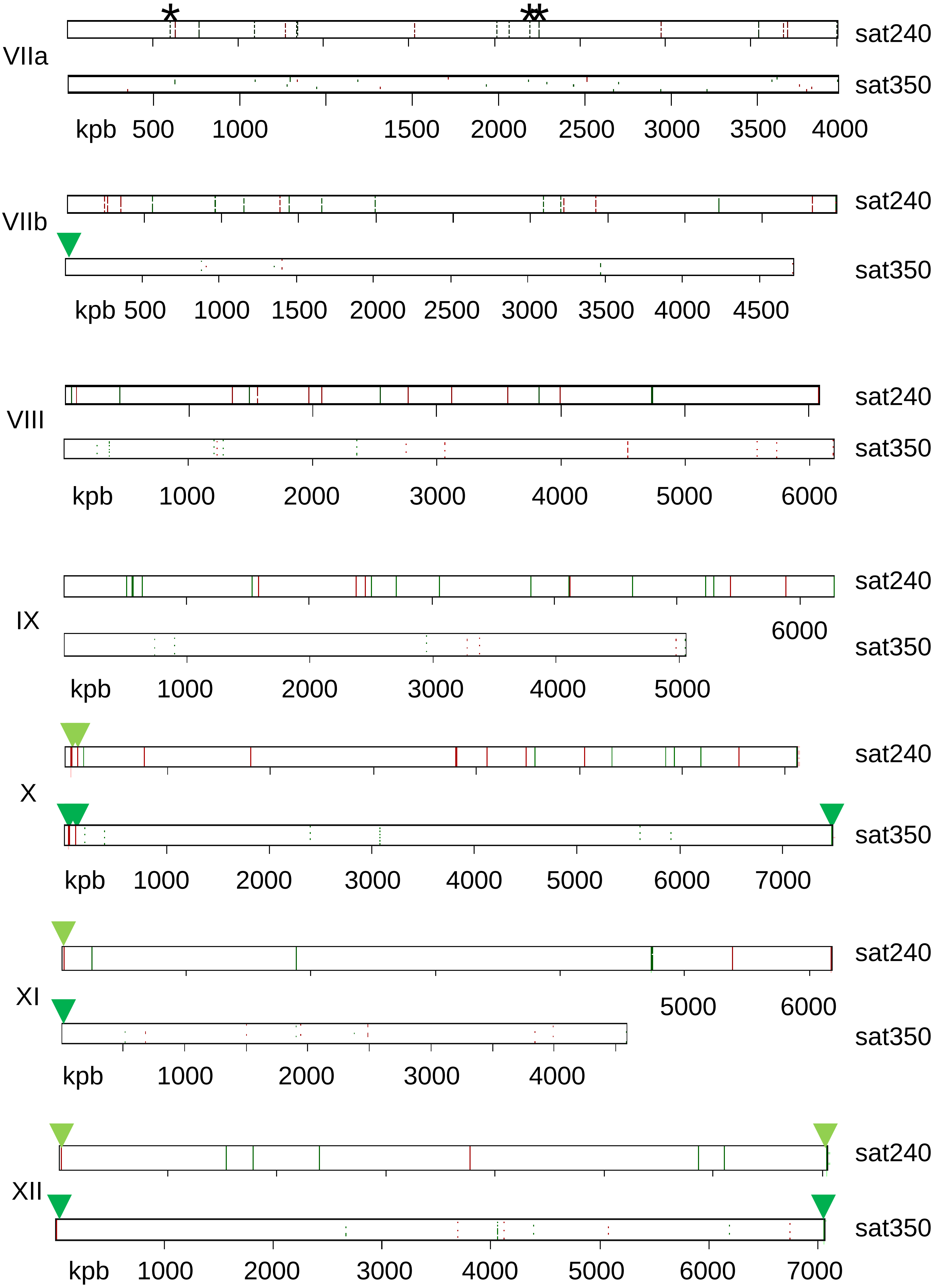

VIIa
sat240
sat350
kpb 500 1000 1500 2000 2500 3000 3500 4000
VIIb
sat240
sat350
kpb 500 1000 1500 2000 2500 3000 3500 4000 4500
VIII
sat240
sat350
kpb 1000 2000 3000 4000 5000 6000
IX
sat240
6000
sat350
kpb 1000 2000 3000 4000 5000
X
sat240
sat350
kpb 1000 2000 3000 4000 5000 6000 7000
XI
sat240
5000 6000
sat350
kpb 1000 2000 3000 4000
XII
sat240
sat350
kpb 1000 2000 3000 4000 5000 6000 7000

Figure S3

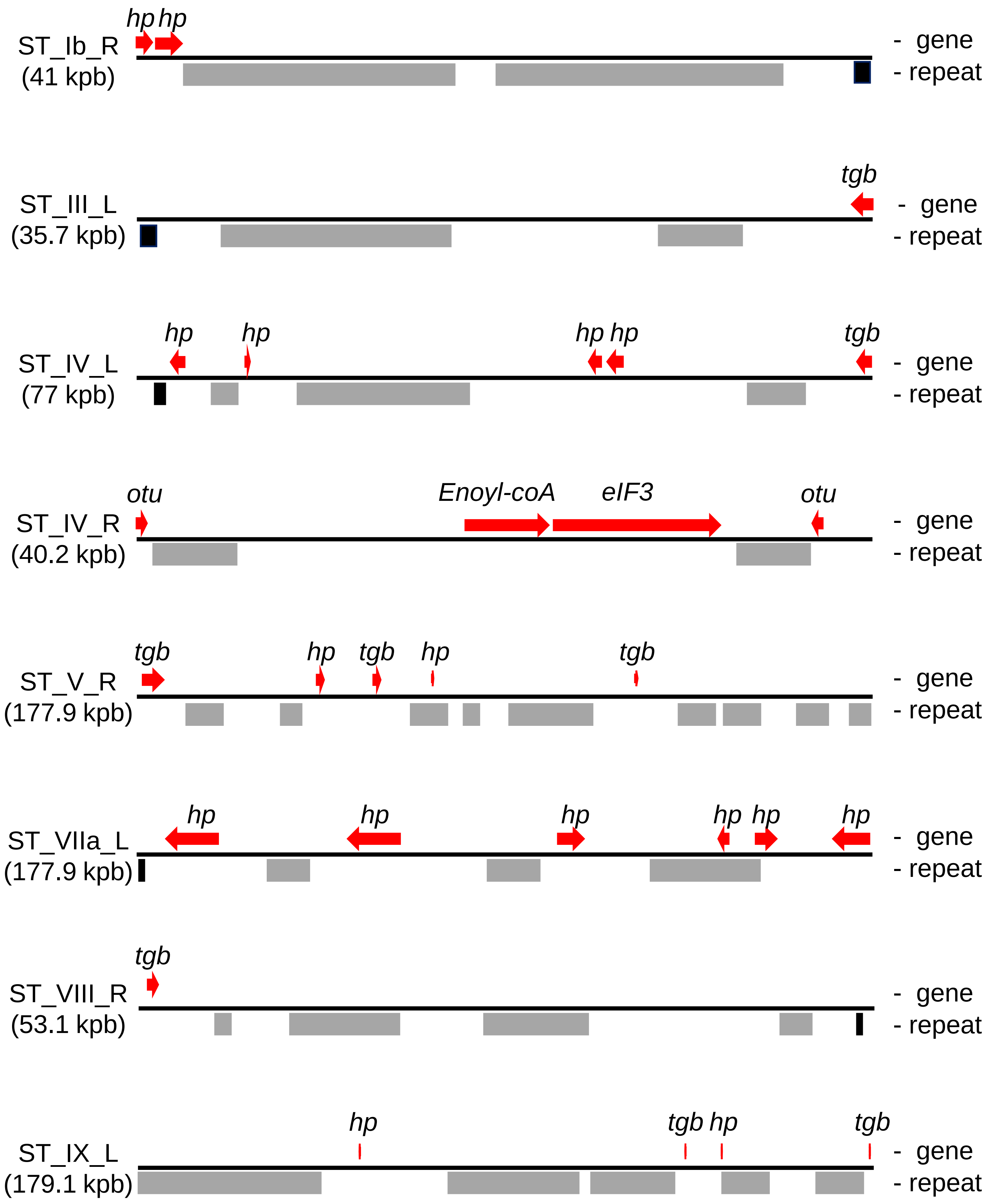
hp hp
ST_Ib_R
(41 kpb)
tgb
ST_III_L
(35.7 kpb)
hp hp hp hp tgb
ST_IV_L
(77 kpb)
otu Enoyl-coA eIF3 otu
ST_IV_R
(40.2 kpb)
tgb hp tgb hp tgb
ST_V_R
(177.9 kpb)
hp hp hp hp hp hp
ST_VIIa_L
(177.9 kpb)
tgb
ST_VIII_R
(53.1 kpb)
hp tgb hp tgb
ST_IX_L
(179.1 kpb)
- gene
- repeat

Figure S4
